# Unraveling the Role of Shared Vision and Trust in Constructive Conflict Management of Family Firms. An Empirical Study From a Mixed Methods Approach

**DOI:** 10.3389/fpsyg.2021.629730

**Published:** 2021-06-15

**Authors:** Cristina Alvarado-Alvarez, Immaculada Armadans, María José Parada, M. Teresa Anguera

**Affiliations:** ^1^Department of Basic, Developmental and Educational Psychology, Universitat Autònoma de Barcelona, Barcelona, Spain; ^2^Department of Social Psychology and Quantitative Psychology, University of Barcelona, Barcelona, Spain; ^3^Department of Social Psychology and Quantitative Psychology, Faculty of Psychology, PsicoSAO-Research Group in Social, Environmental, and Organizational Psychology, Institute of Research in Education, University of Barcelona, Barcelona, Spain; ^4^Strategy and General Management Department, ESADE Business School, Universitat Ramon Llull, Barcelona, Spain; ^5^Faculty of Psychology, Institute of Neurosciences, University of Barcelona, Barcelona, Spain

**Keywords:** shared vision, trust, constructive conflict management, family firm, mixed methods approach

## Abstract

Family firms are a unique setting to study constructive conflict management due to the influence of family ties of the owning family imprinting a sense of common purpose and shared destiny, and high levels of trust. We study the relationship between shared vision and trust that intervene in the adoption of constructive conflict management. To achieve our purpose, we carried out a systematic indirect observation using a mixed methods approach. We used the narratives of 17 semi-structured interviews, audio-recorded and transcribed, of family and non-family managers or directors from five Spanish family firms in the siblings' partnership stage, combined with documentary data obtained from different sources. Intra- and inter-observer reliability were confirmed. Results show a dynamic relationship between shared vision and specific components of trust (benevolence and ability) at different levels of conflict management. We also provide evidence of specific processes of concurrence-seeking and open-mindedness in family and ownership forums accounting for the relevance of family governance in these type of organizations. Family firms are a sum of several subsystems which exhibit a particular resources configuration. This study sheds light on constructive conflict management in family firms opening interesting avenues for further research and offering practical implications to managers, owners, and advisors.

## Introduction

Organizations are fertile ground for conflicts. They respond to the high demands of a highly changing environment, which exerts many pressures on the teams and demands people to solve their dissents, effectively collaborate, and make agile decisions (De Dreu and Gelfand, 2008). Conflict is a multilevel (e.g., individual, teams, organizational, culture) phenomenon (Lewicki and Spencer, 1992; De Dreu and Gelfand, 2008), which poses unique challenges to organizational life. Current evidence supports both the negative and positive effects of conflicts (e.g., Jehn and Mannix, 2001; Spector and Bruk-Lee, 2008). On the one hand, conflict may bring harmful consequences like stress, absenteeism, and turnover (Spector and Bruk-Lee, 2008). On the other hand, conflict can drive innovation, change, and enhanced personal relationships in the workplace when it is constructively managed (Tjosvold et al., 2014; Elgoibar et al., 2016; Mikkelsen and Clegg, 2018). In this matter, trust and open-mindedness play a critical role in displaying its constructive potential (Tjosvold et al., 2014; Elgoibar et al., 2016).

Although the study of conflict in organizations has a long trajectory (e.g., Jehn, 1997; Jehn and Mannix, 2001; De Dreu and Gelfand, 2008), some questions are still pending. Given that conflict is a relational process which emerges in a context “with a sense of history, a normative trajectory, and changing circumstances” (Coleman and Kugler, 2014, p. 963), the exploration of a unique context, such as family firms opens attractive doors to new research in the field of conflict management, given their uniqueness emanating from the overlap of two social systems (the family and the business) (Lansberg, 1983) and the overlapping roles of family members in different decision making domains. Precisely, scholars highlight the need to support with empirical evidence the primary roots of constructive conflict in this context (e.g., Alvarado-Alvarez et al., 2020). Specific contexts as family firms would shape organizational processes like conflict management in a different way (Ployhart and Hale, 2014).

Family businesses are a mounting portion of enterprises across the world. Only in Europe, family firms represent 70–80% of private companies and account for about 40–60% of employment (Botero et al., 2015). The economies of many countries mostly depend on family enterprises (Basco, 2015; Memili et al., 2015). In essence, a family business is: “a business governed and/or managed with the intention to shape and pursue the vision of the business held by a dominant coalition controlled by members of the same family or a small number of families in a manner that is potentially sustainable across different generations of a family or families (Chua et al., 1999, p. 25).” Therefore, families in business are a valuable type of resource that must be preserved and promoted as primary assets (Aldrich and Cliff, 2003), which can derive in a key competitive advantage for economies.

Families in business can be considered “the brain and the heart” of this organization type. The brain because they bring direction and a sense of destiny to their companies (Chua et al., 1999) through critical processes as decision-making and strategic behavior (Chrisman et al., 2005; Sharma et al., 2014), and the heart because families inspire and move their companies through sharing family history, values and emotions (Bee and Neubaum, 2014). Therefore, the uniqueness of family firms undoubtedly resides in family resources (Habbershon and Williams, 1999), which represent both advantages and challenges in areas, such as conflict management (Pieper, 2010; Alvarado-Alvarez et al., 2020), family governance (Suess-Reyes, 2017), and innovation (Carnes and Ireland, 2013) among others.

As mentioned earlier, the family's presence in the business affects its entirety, imprinting a sense of common purpose and shared destiny (Miller, 2014; Neff, 2015). The high levels of trust among family members would contribute to build-up lasting and flourishing organizations (Sundaramurthy, 2008; Cater and Kidwell, 2014; Eddleston and Morgan, 2014) and convert these groups into fertile ground for constructive conflict. However, most of the literature points out the dark side of conflict in family firms given the co-existence of both family and business logics, which may open the door to controversies (Reay et al., 2015) and the demise of the family business (Großmann and von Schlippe, 2015) if they are not constructively managed (Alvarado-Alvarez et al., 2020). The higher complexity of family firms makes them more prone to conflict (Lansberg, 1983; Davis and Harveston, 2001; Pieper, 2010). Therefore, research has paid less attention to the bright possibilities offered by constructive conflict in family firms (Alvarado-Alvarez et al., 2020).

Current evidence shows that conflict may play a positive role in the continuance of family firms by triggering change (Sharma et al., 1997; Claßen and Schulte, 2017) and innovation (De Clercq and Belausteguigoitia, 2015; Kammerlander et al., 2015). Challenging conventional wisdom, a conflict can be “a constructive force” (Mikkelsen and Clegg, 2018, p. 3) that contributes to the long-term development and sustainability of family firms.

Moreover, some last reviews encourage exploring both the negative and positive sides of conflict management (Caputo et al., 2018; Qiu and Freel, 2020). Evidence from organizational psychology shows that family expectations are not a significant source of stress and work-family conflict (Beehr et al., 1997). These authors (Beehr et al., 1997) alluded to the characteristics of the samples of prior studies in the field (e.g., based on advisors; Beehr et al., 1997) bias the understanding of the family side as a source of conflict. Indeed, the use of an ambiguous definition of conflict (Tjosvold, 2008; Frank et al., 2011; Alvarado-Alvarez et al., 2020) may also deviate the attention of the scholars to adverse outcomes of conflict (Qiu and Freel, 2020).

These factors make necessary a more fine-grained conceptualization of conflict, which sees the phenomena through the lens of constructive conflict management (e.g., Tjosvold et al., 2014; Elgoibar et al., 2016). Aligned to this aim, a conceptual work recently published has proposed that both components of shared vision and trust may be considered as cognitive and relational roots of constructive conflict management dynamics in family firms (Alvarado-Alvarez et al., 2020).

In summary, given that the context of family firms has been under-explored in organizational psychology (Gagné et al., 2014), it offers interesting opportunities to explore group processes, such as conflict (Frank et al., 2011; Loignon et al., 2015; Caputo et al., 2018). Recent works try to bridge the gap between psychology and family business research (e.g., Jiang et al., 2018; Strike et al., 2018; Kammerlander and Breugst, 2019). In response to these gaps, this research aims to refine our understanding of the interplay between shared vision, trust, and constructive conflict management in family firms.

In the following sub-sections, we explain the theoretical framework and the relevance of studying these three variables: shared vision, trust, and constructive conflict management. Then the paper continues with the methods and data analysis. Finally, the article ends with the discussion and conclusions sharing contributions, limitations and further research.

### Shared Vision

In the literature of organizational psychology, a shared vision has been defined “as a common mental model of the future state of the team or its tasks that provides the basis for action within the team” (Pearce and Ensley, 2004, p. 260–261). In the context of family firms, shared vision represents an optimistic view of the future, also known as 'family dreams' (Boyatzis and Soler, 2012). It is a set of goals and purposes which energize the group and promote change in organizations. This view of the future creates a sort of emotional contagion atmosphere in the group (Boyatzis et al., 2015). The shared vision is also an expression of the workplace's relational climate (Boyatzis and Rochford, 2020) and a needed team functioning (Marlow et al., 2018). This group's beliefs inspire the group to collaborate toward a common purpose (Lord, 2015).

Shared vision is a component of the unique organizational culture of family firms, which has a significant positive impact on business performance because it behaves as a driver to achieve the organizational future (Neff, 2015). Some elements are distinctive of shared vision in family firms. Usually, it includes family and business purposes (Knapp et al., 2013). It is deeply rooted in family past experiences and condensates the main learnings and insights into the family business history (Jaskiewicz et al., 2015). It also has a transgenerational orientation (Knapp et al., 2013; Jaskiewicz et al., 2015; Diaz-Moriana et al., 2020). It is rooted in the business founders' history (Lord, 2015), but the following generations also participate in its development. It is not a fixed picture. It is considered a relevant factor because a shared vision promotes emotional bonding between family members in the context of high family influence oriented to business continuity (Wang and Shi, 2020).

In this sense, family narratives would contribute to this process (Kammerlander et al., 2015; Parada and Dawson, 2017). Shared vision contributes to the perception of collective commitment, and it is an expression of psychological capital inside the family firm (Memili et al., 2013; Miller, 2014). Indeed, a shared vision would promote enthusiasm in the next generation who will be more committed to entrepreneurial activity (Miller, 2014; Bettinelli et al., 2017). For instance, the selection of a daughter as a successor can be predicted if this person shares a future vision of the business with their parents (Overbeke et al., 2015).

Besides the legacy orientation and business purposes, family harmony norms may also be present in this future frame. In this sense, Kidwell et al. (2012, p. 507) argue that “norms of family harmony help to focus the efforts of family members on the success of the firm, reinforcing the idea of a team-based ethical climate in which family members cooperate with one another.” These expectations about family harmony directly connect to constructive conflict management in a way that a shared vision would lead family members to perceive conflict as an opportunity and “a driver of change” (Claßen and Schulte, 2017, p. 1204). At the same time, this collective purpose would promote open-mindedness and the group's learning capacity (Miller, 2014; Lord, 2015).

Some works highlight the positive influence of shared vision on innovation through having a good impact on collaboration (Bigliardi and Galati, 2018), new product development (Cassia et al., 2012), and strategic flexibility (Craig et al., 2014). A shared vision as a collective cognition between family and non-family members would enhance innovation (Madison et al., 2020). A relevant issue to address in this research is to refine our understanding about the relationship between shared vision, trust and constructive conflict management in the context of family firms.

### Trust

Organizational development is rooted in collaboration and trust (Whitener et al., 1998; Elgoibar et al., 2016). Trust promotes interdependence, given that it represents an expectation about goal facilitation (Tjosvold et al., 2016). In other words, if we expect that another person will help us achieve our goals, we will probably trust him/her, making cooperation a more likely case. Trust is an expression of “confident positive expectations regarding another's conduct” (Lewicki et al., 1998, p. 439).

At the same time, trustworthiness connects with our sense of being vulnerable to the actions of the other person (Mayer et al., 1995). In this context, perception of ability, benevolence, and integrity would create and sustain trust in organizations (Schoorman et al., 2007). It means that managers influence upon the basis of their skills and competences (Mayer et al., 1995; Schoorman et al., 2007). If they are perceived as competent “to manage the task at hand” (Stedham and Skaar, 2019, p. 4), teams will trust this person. Integrity refers to the perceived consistency between words and actions (Mayer et al., 1995; Schoorman et al., 2007; Stedham and Skaar, 2019). A perception of caring, genuine concern with others' needs, and benevolent motives contribute to trust (Mayer et al., 1995; Stedham and Skaar, 2019).

McAllister (1995) distinguishes two types of interpersonal trust: cognition and affection-based trust. It means that we trust based on having “good reasons” (McAllister, 1995, p. 25). Also, the emotional ties between individuals are a source of trust (McAllister, 1995). The three components of ability, integrity, and benevolence (Mayer et al., 1995) are foundations of both cognition and affection-based trust (McAllister, 1995). We may have “good reasons and feeling an emotional connection” to trust on people whom we perceive capable (cognitive), upright (both cognitive and affective), and benevolent (affective) (Lewicki and Brinsfield, 2017). It seems logical that in family firms, the different components of trust (Mayer et al., 1995) are also relevant, and they would show some differences depending on the family or business roles (Knapp et al., 2013).

In the context of family firms, the perception of similarity rooted in the family history shared values and goals would be an essential source of trust (Identity-based trust; Lewicki, 2006). The expectations about the ownership rules or the compliance with the constitution of the family would also steam trustworthiness (Calculus-based trust; Lewicki, 2006).

Trustworthiness creates the right conditions for the emergence of a constructive conflict management in organizations, which is also beneficial for trust relationships (Tjosvold et al., 2014). In a trustful context, people feel safe to express their views openly, discuss controversies and put their efforts to integrate this exchange in solutions mutually agreed upon because they understand that everything is beneficial in order to achieve their mutual goals (Tjosvold et al., 2014).

When applying this theory to the context of family firms, current evidence points out that we should consider some specificities regarding this mutual influence process, given that trust stems from family ties to a great extent (Alvarado-Alvarez et al., 2020). The family nexus would create high vulnerability and affection-based trust (Mayer et al., 1995; McAllister, 1995), which creates a fertile ground for collaboration and constructive conflict management (Alvarado-Alvarez et al., 2020). We may hypothesize that a shared vision would promote higher cognition and affection-based trust between family members (McAllister, 1995).

Family involvement is a source of social capital (Pearson et al., 2008) being trust an essential component of organizational psychological capital in family firms (Meier and Schier, 2016). This bundle of resources or familiness (Habbershon et al., 2003) reports competitive advantages to firms with a top management composed of family members (Pearson et al., 2008) who share high levels of trust (Cabrera-Suárez et al., 2015). Trust catalyzes interaction between leaders and collaborators (Wang and Shi, 2020) and minimizes dysfunctional conflict (Sundaramurthy, 2008).

Trust in family firms adopts different ways according to their life cycle (Sundaramurthy, 2008). In the initial stages, trust stems from the founder and adopts a particularistic view. As the company grows, trust shifts to an institution-based view (Wang and Shi, 2020).

There is evidence that trust has a positive effect on the performance of family firms. Specifically, enhancing innovation (Calabrò et al., 2018). In this sense, social capital and a positive emotional climate boost innovation (Sanchez-Famoso et al., 2014; Bernoster et al., 2018; Daspit et al., 2019). Trust in the family firm's collective abilities also increases innovation (Holt and Daspit, 2015). Indeed, trust relationships with the external stakeholders also contribute to innovation by promoting honest feedback in evaluating innovation projects (Frank et al., 2019).

It makes sense that given that innovation involves taking risks to express divergent opinions (sometimes contrary to the majority position) and assuming that novel ideas may fail (Johnson, 2015), trust would play an essential role in constructive conflict and innovation in family firms. However, there is evidence that some trust-breaching practices, such as asymmetry in the accountability of incentive norms impair the innovation of family firms (De Massis et al., 2016). A study about innovative successful practices of family enterprises identified trust and constructive conflict as mutually reinforced processes in the innovation process (Frank et al., 2019).

Although there is evidence that trust is present across the multiple levels of family firms (Eddleston and Morgan, 2014), trust merits further research in this unique context (Eddleston et al., 2010; Wang and Shi, 2020). Responding to this call, one of the aims of this research is to explore and understand the role of trust in constructive conflict management in family firms.

### Constructive Conflict

Conflict is natural and pervasive in interpersonal relationships as an old said state, “conflict is the spice of life” (Lewicki and Spencer, 1992). Like spices in cooking, conflict elicits a variety of responses, diverse emotions and experiences. It is intrinsic to social interaction because people have different goals, ideas, and activities. Diversity by itself does not cause conflict; it is more common when the parties involved perceive it as a source of incompatibility, interference, and negative emotions. Simultaneously, diversity creates better cooperation conditions in teams (Kozlowski and Chao, 2012).

This research finds inspiration in Social Interdependence Theory, which has been considered one of the five most influential organizational conflict management approaches (Coleman et al., 2012). The most representative work is the seminal theory of cooperation and competition developed by Morton Deutsch in the late forties (Deutsch, 1994). According to Deutsch's assumptions, people who depend on each other to accomplish their goals are prone to conflict (Deutsch, 2011). It is precisely this sense of interdependence that will determine if conflict takes a constructive or destructive course (Deutsch, 2011). Positive interdependence is related to cooperation and rooted in the perception of “similarity in beliefs and attitudes, a readiness to be helpful, openness in communication, trusting and friendly attitudes, sensitivity to common interests and deemphasis of opposed interests, an orientation toward enhancing mutual power rather than power differences…” (Deutsch, 1994, p. 112). A cooperative approach leads to constructive conflict management, reporting positive outcomes as personal well-being, improving relationships, or innovation, among others (Tjosvold et al., 2014).

Under this approach, the participants emphasize “on mutual goals, understanding everyone's orientation” toward mutual benefit, and incorporating several positions to find a solution right for all (Alper et al., 2000, p. 629). Cooperation and constructive conflict can unleash the best organizations and teams, reporting optimal outcomes for groups and individuals regarding sustainability and innovation (Elgoibar et al., 2016). Open-mindedness is a central process of constructive conflict. The main research gaps detected about this process in the family business field will deserve our attention in the following sub-section.

### Open-Mindedness

Open-mindedness is the foundation of constructive conflict in organizations (Tjosvold et al., 2014), also known as constructive controversy (Tjosvold et al., 2014; Johnson, 2015). According to Tjosvold et al. (2014), “open-minded discussion occurs when people work together to understand each other's ideas and positions, impartially consider each other's reasoning for their positions, and seek to integrate their ideas into mutually acceptable solutions” (p. 549). Open-mindedness involves a critical assessment of options, beliefs, and receptivity to exploring all the alternative courses of action (Lord, 2015). Compared to other cooperative approaches, such as concurrence-seeking (Johnson, 2015), open-mindedness or constructive controversy is related to more creativity, a deeper understanding of the issues (Johnson, 2015), and a higher sociomoral climate (Seyr and Vollmer, 2014).

To our understanding, open-mindedness in family firms is an underexplored topic. Some studies have approached conflict management phenomena in family firms (e.g., Sorenson, 1999; Sciascia et al., 2013; De Clercq and Belausteguigoitia, 2015), but there are still intriguing avenues to explore. Mostly, concerning the possibilities offered by constructive conflict so that family firms may release all their potential (Alvarado-Alvarez et al., 2020). Some inquiries about the uniqueness of open-mindedness in family firms deserve further attention.

In business-related conflict, open-mindedness would be more frequent, especially when discussing non-family managers/directors. The mix between family and ownership roles might favor this cooperative approach. The use of particular conflict management strategies depends on the roles, the issues to discuss, and the expected outcomes. Concurrence seeking may be more used than open-mindedness to manage family-related conflict, given that families in business tend to avoid conflict (Danes et al., 2000).

Family members' concerns and expectations regarding family harmony may affect the adoption of open-mindedness. For instance, survey studies reported that collaboration showed better outcomes for both families and businesses. Meanwhile, compromise and accommodation contributed to positive family outcomes (Sample of study: 59 companies; Sorenson, 1999). Expecting to achieve better outcomes for family relationships, family firms may prioritize achieving consensus in detriment of open discussion. Besides, they may avoid an appraisal of alternative ideas and action courses to prevent conflicts between family members. The majority position may use pressure to achieve agreement (Johnson and Johnson, 2009; Johnson, 2015).

Open-mindedness would report several benefits for family firms. Theoretically, a conversation orientation would enhance innovativeness (Sciascia et al., 2013). Innovativeness understood as “a firm's tendency to engage in and support new ideas, novelty, experimentation, and creative processes that may result in new products, services, or technological processes” (Lumpkin and Dess, 1996, p. 142).

When two or more generations participate in the process, the entrepreneurial orientation is reinforced (Chirico et al., 2011). A learning orientation (being shared vision and open-mindedness two of their dimensions) enhances entrepreneurial orientation according to a survey carried out with 509 Spanish SMEs (Hernández-Linares and López-Fernández, 2018).

Open-mindedness would be present at different levels, such as the top management team and governance forums (Bettinelli, 2011; Rosenkranz and Wulf, 2019), where family members are involved, sometimes with overlapping roles in the different areas. In the end, open-mindedness would contribute to creating a shared vision and increasing trust, reporting unique advantages in terms of innovativeness (Lambrechts et al., 2017). Board openness (Kanadli et al., 2020) and knowledge sharing (Cunningham et al., 2016) are beneficial for unleashing the constructive effects of conflict in family business performance. Moreover, information exchange moderates the relationship between the diversity of top management teams and organizational outcomes (Ling and Kellermanns, 2010).

However, family firms also need to overcome some barriers to open-mindedness like excessive parental altruism (Lubatkin et al., 2005; Kidwell et al., 2018), authoritarian power styles (Mussolino and Calabrò, 2014), founder centrality (Kammerlander et al., 2015), rigid family roles (Friedman, 1991), and hidden agendas (Pieper et al., 2013). Under these circumstances, family firms recur to third parties to manage conflict (Lewicki et al., 2016; Qiu and Freel, 2020). Third parties may be formal (e.g., consultants), informal (e.g., spousal, friends), or members of the family business board (Strike, 2012). Third parties may assume different roles in the process of conflict management (Lewicki et al., 2016). Among the main ones, there is the control of the process (e.g., mediators of conflicts) or decision arbitrators (not necessarily as formal arbitrators), playing an autocratic role (Goldman et al., 2008) and process consultation (Lewicki et al., 2016). The last might have a relevant role in constructive conflict, given that one of their goals is to assist the participants of the conflict in improving communication (Lewicki et al., 2016). According to Deutsch (1994), third parties should help participants change their destructive conflict patterns and engage a joint problem-solving approach.

**Figure 1:**
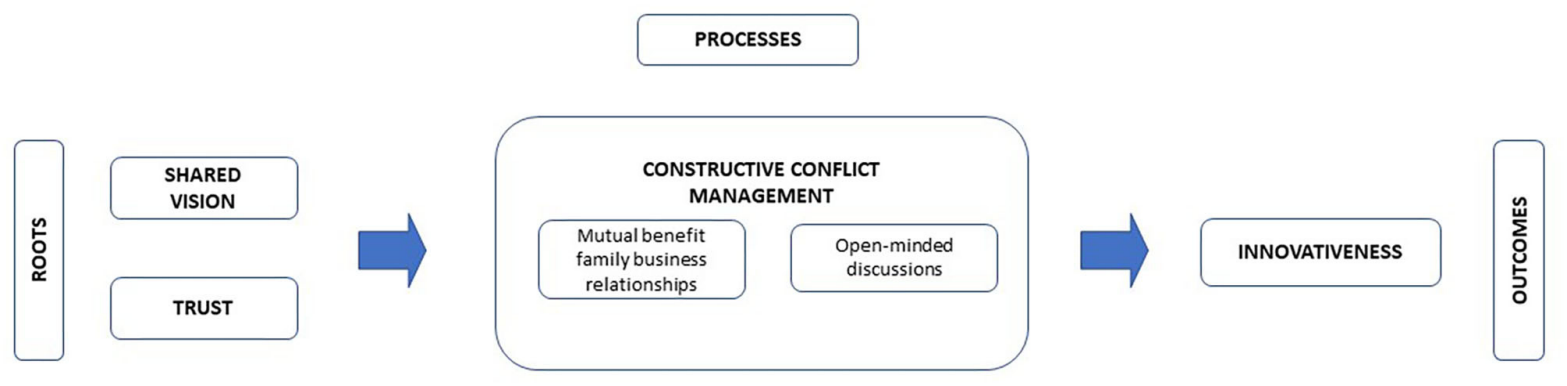
Theoretical model of this research. Source: Adapted from Alvarado-Alvarez et al. (2020).

In summary, current evidence calls for further research on the possible influence of shared vision, trust, and open-mindedness in developing constructive conflict dynamics in family firms (see Figure 1). This is what we study in this paper.

## Materials and Methods

In the following sub-sections, we explain the design, participants, instruments, and procedures used to achieve our research aims.

### Design

This research is in the intersection between mixed method and case study research (Plano Clark et al., 2018; Walton et al., 2019). According to Plano Clark et al., a mixed method case study is “a research approach that intentionally intersects the assumptions, intents, logics, and methods of mixed methods research and case study research in order to more completely describe and interpret the complexity and theoretical importance of a case or cases” (2018, p. 20). This study is an example of nesting the mixed methods within a case study research because it “employs a ‘parent’ case study design and uses mixed methods by collecting, analyzing, and integrating qualitative and quantitative data” (Guetterman and Fetters, 2018). In family business research, a case study mixed methods research is an appropriate option to advance current knowledge (López-Fernández and Molina-Azorin, 2011; Reilly and Jones, 2017; Walton et al., 2019).

Separately, each type of research design reports interesting opportunities. For the side of the case study research, it “allows investigators to focus on a case and retain a holistic and real-world perspective, such as in studying individual life cycles, small group behavior, organizational and managerial processes” (Yin, 2015, p. 4). According to this author, case studies allow addressing “how” and “why” questions. They involve “an intensive study of a single unit for the purpose of understanding a larger class of (similar) units…observed at a single point in time or over some delimited period of time” (Gerring, 2004, p. 342).

Case studies are an excellent option to understand social complex phenomena by applying and extending current theories (George and Bennett, 2005). Indeed, these authors identify four advantages of case studies: “their potential for achieving high conceptual validity; their strong procedures for fostering new hypotheses; their value as a useful means to closely examine the hypothesized role of causal mechanisms in the context of individual cases; and their capacity for addressing causal complexity” (George and Bennett, 2005, p. 19).

Gerring (2004, p. 341) asserted that “The case study survives in a curious methodological limbo.” Although the case study is not exempt of controversies, we considered the enormous attractive of using a multiple case design. The case is a polyhedric entity characterized by high levels of complexity where there are different orbits interacting as can be family and business.

A multiple case design is not an aggregation of single cases; instead, it involves detecting the structure and patterns of a single case and testing if the following cases share a similar structure (Anguera, 2018). In this sense, cross-case comparisons allow a more fine-grained understanding of common and unique traits of each case (Guetterman and Fetters, 2018). Case studies also involve dealing with several trade-offs, such as the selection bias, the balance between parsimonious and rich data, and the sacrifice of generalizability over internal validity (George and Bennett, 2005), among others. Apart from the existing typologies (Stake, 1994; Thomas, 2011; Yin, 2014), the logic of the single case is intra-case by nature (Hilliard, 1993), and for this reason, some certain conditioned behaviors were proposed for each case, depending on the objectives of the study, but the method applied to detect multiple cases has been to start from parallel relationships between focal and conditioned behaviors, understanding that these relationships have been obtained quantitatively through a robust analysis.

Designing a case study mixed methods research involves making some decisions about the qualitative and quantitative components. For the qualitative component in this study, we employed the narrative approach, which involved collecting narratives from different case participants by gathering their stories and reports about individual experiences (Creswell, 2013). The sources of these narratives were basically in-depth interviews. The quantitative component was addressed by conducting a polar coordinate analysis of the matrix of codes obtained using an *ad hoc* indirect observation system to process these narratives (See Supplementary Material—The Indirect Observation System Handout). After obtaining the matrix of codes of each case and applying the polar coordinate analysis, we compared the structures of associative relationships that were statistically significant to select those in which at least three or more cases coincided. The reasoning behind this election was that we were interested in detecting the presence of similar structures as an empirical evidence of suitability and validity of the theoretical model (Alvarado-Alvarez et al., 2020).

The decision about how many parallel results of the different unique cases must coincide for us to consider a multiple case is conventional, and is not logically fixed in the literature. In this sense, Sandelowski (1996, p. 527) said: “The appropriate initial approach to any kind of qualitative data analysis is to understand and treat each sampling unit as a case, whether that is defined empirically (e.g., as a certain person or family or event) or analytically (e.g., by a diagnostic or other theoretical, constructed, or researcher-invented category) before looking for commonalities and differences across cases. The analyst works to discern what elements comprise the case and, more importantly, the way they come together uniquely to characterize the case.” A quarter of a century later we have been accumulating evidence from studies in which multiple cases have been detected, and the aforementioned conventionality is maintained.

**Figure 2:**
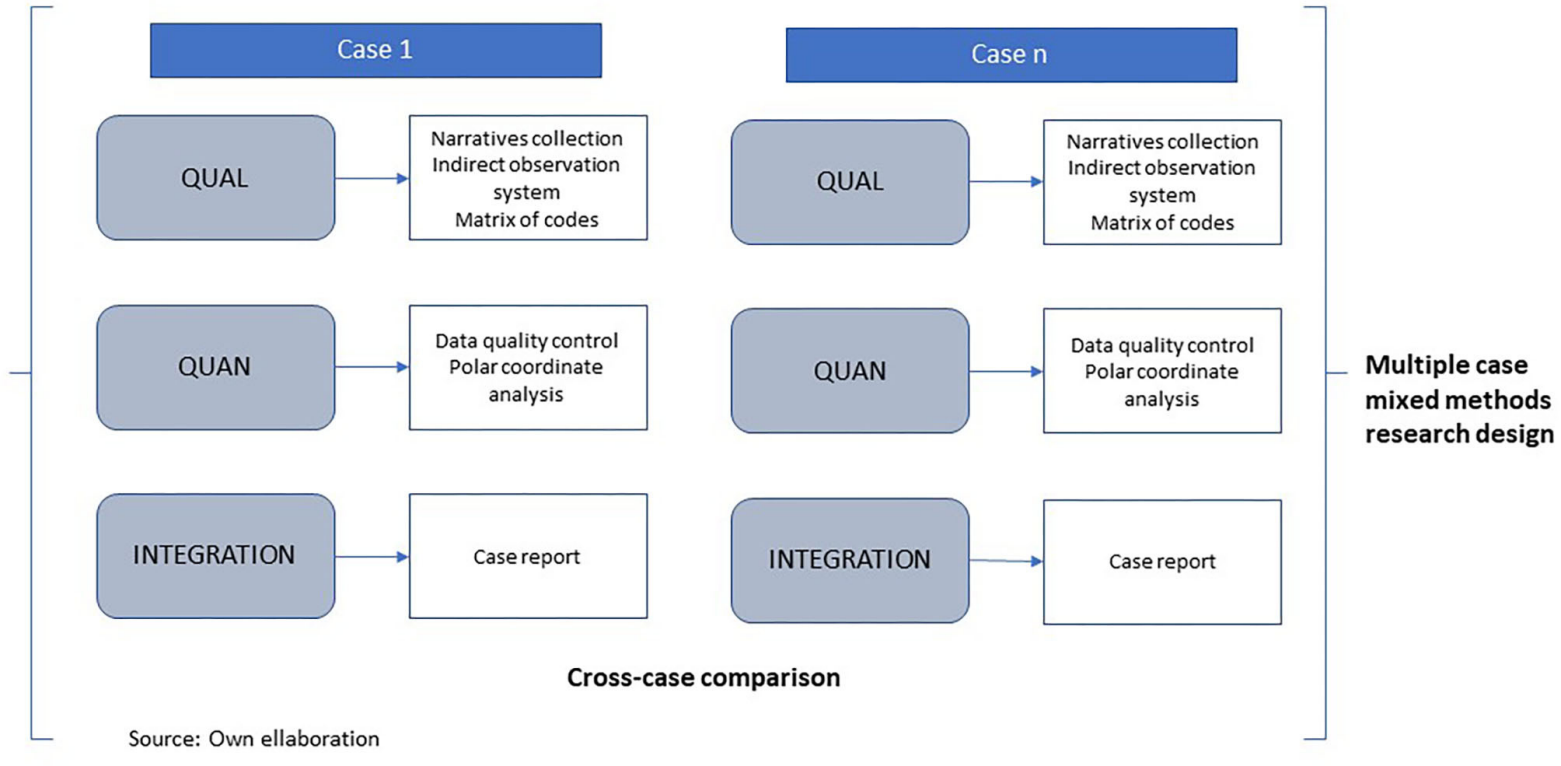
Research design. Source: Own elaboration.

Thus, the research design involved three phases: (1) Qualitative (QUAL) consisted of collecting narratives, coding by an indirect observation system, and converting it into a matrix of codes. (2) Quantitative (QUAN): we conducted a polar coordinate analysis to quantify the narratives. (3) Qualitative (QUAL) to integrate qualitative and quantitative findings (see Figure 2).

The following sub-section describes the indirect observation methodology employed to conduct the mixed methods study.

### Indirect Observation

Observational studies allow observing psychological phenomena in the natural context. In the last two decades, Anguera et al. have built a body of robust evidence regarding the use of observation in a variety of contexts supported by a “highly systematic data collection and analysis, stringent data quality controls, and the merging of qualitative and quantitative methods” (Anguera et al., 2018, p. 3). An extension of the observational methodology is the indirect observation to analyze textual material and narrative data from verbal behavior or documentary sources (see Anguera et al., 2018, for more detail).

Thus, this study adopts an indirect observation method (Anguera et al., 2018) to grasp the nuances of the reciprocal relationship between shared vision, trust and constructive conflict management in family firms. Under a mixed methods approach, we systematically observed the textual material obtained from open-ended interviews using an *ad hoc* indirect observation system (Anguera et al., 2018) developed by the authors (See Supplementary Material—the Indirect Observation System Handout). This *ad hoc* instrument was built upon the conceptual framework about constructive conflict management in family firms (Alvarado-Alvarez et al., 2020). The use of indirect observation contributes to systematizing and achieving more rigor in the study of narratives (Anguera et al., 2018), a claim in family business research (Wright and Kellermanns, 2011). Moreover, it opens some avenues to study conflict management as experienced by participants in everyday life, which is highly appropriate in order to explore such sensitive issues (Jehn and Jonsen, 2010).

Specifically, we conducted an indirect observation of the narratives collected to systematize the detection process and describe the structure of patterns of behavior (Anguera et al., 2018; Anguera, 2020). This process involved several steps to quantifying the qualitative data (Anguera et al., 2018), which allowed rigor, flexibility, and reduced the loss of the relevant information (Anguera, 2020). The use of this methodological approach has been recognized as highly valuable to explore family phenomena (Plano Clark et al., 2008) as well everyday practices of individuals and groups (Anguera et al., 2018) and communicative processes (e.g., García-Fariña et al., 2018; Del Giacco et al., 2019, 2020; Anguera et al., 2020). Additionally, mixed methods reported several advantages in addressing complex research questions and collecting robust evidence (Yin, 2015).

In studies of indirect observation, it is needed to make decisions about the characteristics of the design (Anguera et al., 2018) considering the number of units of study (one vs. various), the temporality of the records (punctual vs. follow-up), and the level of response (one or multiple dimensions) (Blanco-Villaseñor et al., 2003). In this research, the use of five family firms as units of study, temporal series of events in the life of these units, and the interest in observing several dimensions (shared vision, trust, and conflict management), led to the use of a nomothetic, follow-up and multidimensional study (Blanco-Villaseñor et al., 2003; Sánchez-Algarra and Anguera, 2013).

### Participants

The data collected consisted of 17 in-depth interviews with family and non-family managers (Total of hours: 18.85; see Table 1) and documentary data gathered from several sources (e.g., participants, company's web page, business reports, internet, press media, social networks). The interviews were open-ended although some questions were considered as a reference for assuring that the object of study was explored (See Supplementary Material for see the questions guide used by interviewer). This process was carried out between November 2018 and December 2020. The original language of the interviews was Spanish.

**Table 1:** Characteristics of the participants.

**Case**	**Industry**	**Profile's interviewee**
1	Retail	Founders (mother and father) three sisters two non-family managers
2	Services	Two sisters/ third generation-member
3	Manufacture	Sister
4	Manufacture	Two sisters
5	Services	Son non-family owner non-family manager

Theoretical sampling is a good research strategy to build theories from case studies (Eisenhardt and Graebner, 2007). Therefore, we purposely selected the participants according to the following criteria: (1) An enterprise or family business group owned by a group of siblings. (2) The company was recognized as highly innovative in the industry. (3) The cases were easy to access through the social network of the researchers. All the participants were informed about the objectives of the study, and their consent to participate were also informed to the researchers. A common characteristic of the cases was that they had received family business advising in the last decade (For a thorough description of the cases, see Table 2).

**Table 2:** Case description.

	**Case 1**	**Case 2**	**Case 3**	**Case 4**	**Case 5**
Generation	Second generation	Second generation	First generation	Fourth generation	First generation
Family business history	Founded by an entrepreneurial couple. Siblings have developed their career in the company except one who entered later.	The expansion of the company has been co-led by siblings.	Founded by the father and one of the siblings. It has faced a fast growth in three decades. Sibling group has been working together in this expansion process.	Founded by the grandparents and expanded by an entrepreneurial couple and their children.	Founded by the father. A few years later, a non- family manager co-led the growth and shared the ownership of the company. Children also are participating in management and they have contributed to the fast growth that the company faced in the last decade.
Family involvement	The founder is still involved at the Board of Directors. Sibling group co-lead the company.	Earlier involvement of the successors in the company. Members of the cousin group play managerial roles in the company.	Siblings have been involved since the foundation of the company. Siblings are both at the governance and managerial levels of the company.	Siblings play different roles mainly at the governance level. Next generation is involved in the company.	The founder is still involved at the governance level. Siblings play different managerial roles.
Business complexity (Gimeno et al., 2010)	Medium International Around to 250 employees Partial vertically integrated	Medium Local Around 100–150 hundred employees	High International Around 370 employees Partial vertically integrated	High International More than 200 employees Vertically integrated	High International More than 500 employees Vertically integrated
Ownership	Family	Family	Family	Family +non-family	Family + non-family
Succession	The process of succession in management has been successfully completed.	Effective delegation of managerial roles to successor. Facing the transition from second (sibling partnership) to third generation (cousins consortium). Next generation participates in Family Council.	The generation in charge (sibling partnership) is still young to start a succession process.	The process of succession in governance and management has been successfully completed.	Facing the transition between founder stage and sibling partnership. Next generation participates in the Board of Directors.
Conflict	Family harmony	Family conflict Report of a critical incident related to the participation of the next generation in the company.	Family harmony They report the advantages of having controversies in the Advisory board.	Family harmony In the last years they have invested time and resources in improving family communication between siblings (e.g., coaching services).	Family harmony Report of a critical incident regarding the temporal exit of a child of the company in the past
Governance	Informal meetings Board of directors Family protocol	Family Council Board of directors Advisory Board Family protocol	Family Council Board of directors Advisory board Family protocol	Informal family meetings Family Council Board of directors Family protocol	Family Council Board of directors Family protocol
Innovation	Focused on new markets. Innovativeness committee (recent creation) to trigger internal debates around innovation. Collaborative partnerships with start-ups to explore new product and service development.	Focused on new services and markets. Collaborative partnerships with other companies to improve innovation capacities.	Focused on new internal processes. Innovativeness committee (recent creation) lead by external advisors and managers.	Focused on new markets and services. Innovation through tradition (product development rooted in long-lasting entrepreneurial activity-De Massis et al., 2016). Digitalization.	Focused on new services and internal processes. Recruiting of an external Head of innovation.

### Instruments

#### Recording Instruments

The open-ended interviews were audio-recorded using a mobile application. We performed the verbatim transcription by the Software TRINT. The coding process was carried out by a web application specially designed for this research. It was based on the *ad hoc* indirect observation system. The data model of the web application consisted of a set of master data tables framed on the following entities: table of interviews, observers, participants, companies, units of analysis, and a list of codes organized in dimensions and subdimensions. All of the observations were stored in a coding table.

#### Indirect Observation System

To perform the indirect observation of the narratives, we used an *ad hoc* indirect observation system created by the authors (for a detailed description, see the *Indirect Observation System Handout* in Supplementary Material). The different components of the system were based on the literature review. This indirect observation system consisted of six dimensions: family, business, shared vision, trust, constructive conflict management, and innovation. Most of the dimensions had several subdimensions and categories (see Table 3). The observers were able to use the different dimensions and subdimensions to code the narratives. The categories were mutually exclusive.

**Table 3:** A summary of indirect observation system.

**Dimensions**	**Subdimensions**	**Categories**	**Codes**
Family (1)	Boundaries (A)	Family Non-family Family business External environment	1AFAM 1ANOFAM 1AFAMBIZ 1AEXT
	Generation (B)	Founder Second generation Third generation Fourth generation	1BFOUND 1B2G 1B3G 1B4G
	Family ties (C)	Spousal Parent-children Sibling Cousin Uncle, aunt Niece, nephew Grand-mother Grand-father Grand-children In-law	1CSPOU 1CCHILD 1CSIBL 1CCOUSI 1CUNCL, 1CAUNT 1CNIEC, 1CNEP 1CGRANDM 1CGRANDF 1CGRANDCHIL 1CINLAW
	Family history (D)	Family history	1DFAMHIST
Business (2)	Characteristics (A)	Size Industry Market	2ASIZE 2AINDUST 2AMARK
	Business role (B)	Chief executive officer General manager Chief financial officer Manager Worker Board of directors member Board of advisor member Non-executive role Owner	2BCEO 2BGM 2BCFO 2BMANAG 2BWORK 2BBDME 2BABME 2BNONEX 2BOWN
Shared vision (3)		Shared vision Lack of shared vision	3ASHAVIS 3ALACKSHAVIS
Trust (4)	Trust (A)	Trust	4ATRUST
	Trust components (B)	Ability Integrity Benevolence	4BABILIT 4BINTEG 4BBENEV
	Lack of trust (C)	Lack of trust	4CLACKTRU
	Trust repair (D)	Trust repair	4DTRUREP
Constructive conflict management (5)	Multilevel system (A)	Informal family meetings Family council Managing committee Board of directors Board of advisors	5AFAMEET 5AFACOUN 5AMACOM 5ABOADIR 5ABOAADV 5ACOLLAT 5AOWN 5AFACONST 5ATEAM
		Collaborators Ownership Family constitution Teams	
	Perceived conflict (B)	Perceived conflict Lack of conflict Conflict avoidance	5BCONF 5BLACKCONF 5BCONFAVO
	Types of conflict (C)	Task conflict Relational conflict Work-family conflict	5CTASK 5CRELAT 5CWORKFAM
	Processes (D)	Open-mindedness Close-mindedness Concurrence-seeking	5DOMD 5DCLOSE 5DCONCSEEK
	Third-party assistance (E)	Third-party assistance	5ETHIRD
	Succession (F)	Succession	5FSUCCESS
Innovation (6)	Innovativeness (A)	Innovativeness Collaborative innovation	6AINNOVA 6ACOLLINNOV
	Risk-taking orientation (B)	Risk-taking Risk-avoidance	6BRISKTAK 6BRISKAVOID
	External pressures (C)	External pressures	6CEXT
	Decision-making pace (D)	Agile decision-making Slow decision-making Blocked decision-making	6DAGILE 6DSLOW 6DBLOCK

The family dimension observed the existence of different subsystems around the family system (boundaries), the awareness of the generational stage (generation), the family kinship (family ties), and the references to the milestones of the family history as a group or as individuals. The business dimension allowed coding the narratives regarding the specific attributes (or characteristics) of the company (e.g., size) or its environment or group of companies (e.g., industry or market) and the role played in the organization (business role). The shared vision dimension is concerned with the perception of a “group member's genuine belief that they are working collaboratively toward a common purpose” (Lord, 2015, p. 8) and the image of possibilities, family dreams, and hope (Boyatzis and Soler, 2012). It also allowed observing the perception of the absence of a shared vision between family members (Lack of shared vision). The trust dimension observed the existence of trust (Mayer et al., 1995; Lewicki et al., 1998), the allusions to the different components of trust (ability, benevolence, and integrity), the perception of a lack of trust, and trust repair (Lewicki and Brinsfield, 2017). The dimension of constructive conflict management consisted of six subdimensions: multilevel system, perceived conflict, types of conflict, processes, third-party assistance, and succession. Finally, the dimension of innovation had four subdimensions (innovativeness, risk-taking orientation, external pressures, and decision-making pace) (For a detailed description of the indirect observation system, please see the Indirect Observation System Handout in Supplementary Material).

#### Data Analysis Software

The coding table obtained by the web application was able to export the matrix of codes to Excel. The intra-observer and inter-observer reliability were performed by the Generalized Sequential Querier computer program (GSEQ, v.5.1.23; Bakeman and Quera, 2011). We used the *Tool for the Observation of Social Interaction in Natural Environments* (HOISAN, v. 1.6.3.3.4; Hernández-Mendo et al., 2012) to conduct the polar coordinate analysis, and to draft the vectors with the assistance of the R program (Rodríguez-Medina et al., 2019).

### Procedure

The verbatim of the 17 open-ended interviews was segmented into units of analysis, as suggested by Anguera et al. (2018). The unitizing process involved the division of the textual material into units with meaning (Krippendorff, 2018; Anguera, 2020). A total of 10,442 units of analysis were coded using the web application (see an example of unitizing and coding in Table 4). Before conducting the polar coordinate analysis, the control of data quality was performed (Anguera et al., 2018).

**Table 4:** Some examples of unitizing and coding.

**Units**	**Codes**
Then the differences surfaced	1AFAMBIZ, 1B2G, 1CCOUSI, 1DFAMHIST, 2BCEO, 5BCONF
And in these meetings there was already a more personal, more hurtful issue.	1AFAMBIZ, 1B2G, 1CCOUSI, 1DFAMHIST, 2BCEO, 4CLACKTRU, 5ABOADIR, 5BCONF, 5CRELAT
The discussions were already very strong but mixed with personal issues	1AFAMBIZ, 1B2G, 1CCOUSI, 1DFAMHIST, 2BCEO, 5ABOADIR 5BCONF, 5CRELAT
I try to give my daughters a little support, right?	1AFAM, 1BFOUND, 1CCHILD, 4ATRUST, 4BBENEV
There are some areas that I haven't fully transferred yet but they are secondary areas	1AFAMBIZ, 1BFOUND, 1CCHILD, 5FSUCCESS
The main ones have all been transferred, which is decision-making.	1AFAMBIZ, 1BFOUND, 1CCHILD, 2BCEO, 5FSUCCESS
A constant of first listening and reflecting	1ANOFAM, 1B3G, 1CSIBL, 2BBDME, 4ATRUST, 5ABOADIR, 5DOMD
Because they can tell you something at first	1ANOFAM, 1B3G.1CSIBL, 2BBDME, 5ABOADIR, 5BCONF, 5DOMD
And achieving consensus and agreeing what we are understanding as innovation	1AFAMBIZ, 1B2G, 1CCHILD, 2BBDME, 3ASHAVIS, 5ABOADIR, 5DCONCSEEK, 6AINNOVA
But ¡what is he telling me!	1ANOFAM, 1B3G, 1CSIBL, 2BBDME, 4CLACKTRU, 5ABOADIR, 5BCONF
But hey afterwards, the great capacity of leaving the advise to reflect on saying, well, where are we going?	1AFAMBIZ, 1B3G, 1CSIBL, 2BBDME, 4ATRUST. 5ABOADIR
Because obviously they as a company have a very clear vision that is very different from ours	1AFAM, 1B4G, 1CCOUSI, 3ALACKSHAVIS
They are also very clear that they have to really respect our vision to grow	1AEXT, 1B3G, 1CSIBL, 2BBDME, 3ASHAVIS, 4ATRUST, 4BBENEV, 5ABOADIR
In our case, as we also draw the Strategic Plan together, we are very aligned in everything we do.	1AEXT, 1B3G, 1CSIBL, 2BBDME, 3ASHAVIS, 4ATRUST, 5ABOADIR
They worry about seeing that the plan is being fulfilled	1AEXT, 1B3G, 1CSIBL, 2BBDME, 3ASHAVIS, 4ATRUST, 5ABOADIR

#### Data Quality Control

Given that this is an indirect observation study (Anguera et al., 2018), we conducted both intra-observer and inter-observer reliability. The former consisted of measuring the level of agreement between three observations (in successive weeks; 1, 2, 3) performed by the same researcher of the same units of analysis. The last referred to the level of agreement between three observers in coding the same units of analysis. In both situations, we selected 10% of the textual material (around 1,000 units of analysis) to conduct quality control. For the inter-observer reliability process, three observers were trained for coding. The three observers had different professional backgrounds and levels of research experience, which allowed more rigor in the control of data quality (Anguera et al., 2018). The intra-observer reliability was computed as the average Cohen coefficient (Cohen, 1960) GSEQ (v.5.1.23; Bakeman and Quera, 2011). The average result was 0.69 (good). This result was affected by the fact that the indirect observation system was slightly adjusted between observation 1 and observation 2 (Kappa 1.2 = 0.59; Kappa 1.3 = 0.63; Kappa 1.3 = 0.86; Mean value = 0.69).

The inter-observer concordance was calculated using the same procedure for the intra-observer reliability. The average Kappa coefficient calculated for the level of agreement between the three pairs of observers was 0.65 (good).

### Data Analysis

The polar coordinate analysis is a quantitative analytic technique widely used in observational studies (Arias-Pujol and Anguera, 2017; Portell et al., 2019; Anguera, 2020; Anguera et al., in press). In specific designs, such as multiple mixed methods case, the use of polar coordinates allows conducting the diachronic analysis of the cases (Anguera, 2018; Anguera et al., 2020). The use of polar coordinate analysis reports several advantages. First, it helps investigators to draw conclusions about complex interactions. Second, it exhibits a high predictive value (Maneiro and Amatria, 2018). Third, it allows processing a high amount of textual data (García-Fariña et al., 2018).

This technique was developed by Sackett (1980) and improved by Anguera (1997) through introducing genuine retrospectivity. The polar coordinate analysis is based on the results obtained through the lag-sequential analysis (Bakeman, 1978), including the adjusted residuals (Allison and Liker, 1982). This analysis is conducted at both the prospective, through the positive lag, as well as the retrospective level, through the negative lag that once standardized (Z values) are reduced using the Z_sum_ parameter introduced by Cochran (1954). Z_sum_ is based on the principle that the sum of a number n of independent Z scores (as many calculated prospective as retrospective lags with the same quantity in each case and which should be at least 5) is normally distributed with μ = 0 and σ = 1. The calculation of the Z_sum_ parameter, whose formula is ${\text{Z}}_{\text{sum}}=\frac{\sum Z}{\sqrt{n}}$ (where n stands for the number of lags), allows for the obtention of as many Z_sum_ as lags for each specific category from the prospective and retrospective perspectives. As a consequence, the same quantity of Z_sum_ and lags are obtained for every specific category in each of the two prospective and retrospective perspectives.

Each Z_sum_ may carry a positive or negative sign, which will therefore determine which of the four quadrants will contain the categories corresponding to the conditional behaviors in relation to the focal behavior being displayed. The polar coordinates analysis helps to identify the activation or inhibition relation of the focal behavior and all or some of the categories of the observational instrument, which are the conditional or matching behaviors.

Vectors represent the relationships graphically. The length parameter of the vector ($Lenght=\sqrt{{\left(Z_{sum\;prospective}\right)}^{2}+{\left(Z_{sum\;retrospective}\right)}^{2}}$) and the angle ($\varphi =Arc\;sen\;\frac{Z_{sum\;retrospective}\;}{Lenght}$) are calculated using the Z_sum_ criterium and Z_sum_ matching values for each of the conditional behaviors. For a significance level of 0.05 the length of the vector has to be >1.96. Once the length and the angle corresponding to each vector are obtained, the angle must be adjusted taking into account the quadrant where each vector will be located.

This analysis was carried out through GSEQ software package v.5.1.23 and HOISAN v 1.6.3.3.4. In Table 5, the focal and conditional behaviors selected for analysis are listed. This list was consistent with the dimensions of the study. Therefore, shared vision, trust, constructive conflict management and innovativeness were considered focal behaviors. Family and business dimensions and the remaining set of dimensions of the study were considered conditional behaviors, respectively. In this research, this technique of analysis allowed to describe the structure of reciprocal relationships between the different dimensions of study by capturing the respective activation and inhibition effects shown between narratives. In other words, the activation effect expressed that one dimension activated the presence of the other dimension. Whereas, the inhibition influence meant that the dimension suppressed the presence of the other dimension. Translated to narrative studies means that some narratives appeared together (activation) and some narratives suppressed each other (inhibition).

**Table 5:** List of focal and conditional behaviors selected for analysis.

**Focal behaviors**	**Conditional behaviors**
Shared vision (3)	Family boundaries (1A), Generation (1B), Family ties (1C), and business roles (2B). Trust (4A), components of trust (4B), and lack of trust (4C). Multilevel system (5A), perceived conflict (5B), types of conflict (5C), processes (5D), third-party assistance (5E), and succession (5F). Innovativeness (6A), risk-taking orientation (6B), decision-making pace (6D).
Trust (4A)	Family boundaries (1A), Generation (1B), Family ties (1C), family history (1D), business roles (2B), shared vision (3), multilevel system (5A), perceived conflict (5B), types of conflict (5C), processes (5D), third-party assistance (5E), succession (5F). Innovativeness (6A), risk-taking orientation (6B).
Trust components (4B)	Multilevel system (5A), perceived conflict (5B), types of conflict (5C), processes (5D), third-party assistance (5E), succession (5F), innovativeness (6A).
Lack of trust (4C)	Multilevel system (5A), perceived conflict (5B), types of conflict (5C), processes (5D), third-party assistance (5E), succession (5F).
Perceived conflict (5B)	Family boundaries (1A), Generation (1B), Family ties (1C), family history (1D), business roles (2B), trust (4A), trust components (4B), lack of trust (4C), multilevel system (5A), innovativeness (6A).
Types of conflict (5C)	Family boundaries (1A), Generation (1B), Family ties (1C), family history (1D), business roles (2B), trust (4A), trust components (4B), lack of trust (4C), multilevel system (5A), processes (5D), third-party assistance (5E), succession (5F). Innovativeness (6A).
Processes (5D)	Family boundaries (1A), business roles (2B), trust (4A), trust components (4B), lack of trust (4C), multilevel system (5A), innovativeness (6A).
Succession (5F)	Shared vision (3), trust (4A), trust components (4B), lack of trust (4C).
Innovativeness (6A)	Shared vision (3), Trust (4A), Trust components (4B), lack of trust (4C), perceived conflict (5B), processes (5D), succession (5F).

The polar coordinate analysis has been applied in several fields, as education (García-Fariña et al., 2018; Escolano-Pérez et al., 2019), clinical psychology (Arias-Pujol and Anguera, 2020; Del Giacco et al., 2020), sports (Morillo et al., 2017; Maneiro and Amatria, 2018), environmental psychology (Pérez-Tejera et al., 2018) and health promotion in work (Portell et al., 2019).

## Results

As it was mentioned earlier, we were interested in identifying existing statistically significant associative relationships across most of the cases to account for the existence of a multiple case. For this reason, in this section we describe the results of the polar coordinate analysis of focal and conditional behaviors selected for analysis (see Table 5) were significant associative relationships (vectors with a length >1.96, *p* < 0.05) coinciding in three or more cases (see Tables 6, 7).

**Table 6:** Patterns of coincidence in terms of their associative relationships across the five cases studied.

**Number of coincidences**	**Cases**	**Focal behavior**	**Conditional behavior**	**Quadrant**
Five coincidences	1, 2, 3, 4, 5	Shared vision	Benevolence	III
		Benevolence	Board of directors (multilevel CCM)	III
		Innovativeness	Succession	III
Four coincidences	1, 2, 3, 5	Lack of shared vision	Risk taking orientation	I
			Lack of trust	I
			Perceived conflict	I
		Shared vision	Managing committee	I
		Trust	Collaborators (multilevel CCM)	I
			General manager (business role)	I
		Shared vision	Perceived conflict	III
		Succession	Shared vision	III
	1, 2, 4, 5	Lack of trust	Board of directors (multilevel CCM)	I
		Shared vision	Board of directors member (business role)	III
		Ability	Perceived conflict	III
			Family meetings	III
		Perceived conflict	Ability	III
		Innovativeness	Lack of trust	III
	1, 3, 4, 5	Trust	Innovativeness	III
Three coincidences	1, 2, 3	Lack of shared vision	Family business system (boundaries)	I
		Shared vision	Family business system (boundaries)	I
			Risk-taking	I
			Risk-avoidance	I
			Innovativeness	I
		Ability	External environment (boundaries)	I
		Work family conflict	Family history	I
		Innovativeness	Shared vision	I
		Lack of shared vision	Non-family members	III
		Shared vision	Non-family members	III
		Ability	Ownership	III
		Perceived conflict	Innovativeness	III
			External environment	III
		Open-mindedness	External environment	III
		Innovativeness	Perceived conflict	III
	1, 2, 4	Trust	Succession	I
		Benevolence	Family system	I
		Concurrence-seeking	Family meetings	I
		Open-mindedness	Family meetings	I
			Family council	I
		Succession	Trust	I
		Shared vision	Family system	III
		Ability	Family council	III
			Family system	III
	1, 2, 5	Shared vision	Ownership (multilevel CCM)	I
			Ability	I
		Trust	Managing committee	I
		Lack of trust	Close-mindedness	I
		Ability	Collaborative innovation	I
			General manager	I
			Collaborators	I
			Managing committee	I
		Perceived conflict	Family constitution	I
		Task conflict	General manager	I
		Close-mindedness	Lack of trust	I
		Concurrence-seeking	Ownership	I
		Collaborative innovation	Ability	I
		Shared vision	Family council	III
			Family constitution	III
		Trust	Workers	III
		Ability	Ownership	III
			Workers	III
			Family constitution	III
		Benevolence	Teams	III
		Lack of conflict	Managing committee	III
			Collaborators	III
			Innovativeness	III
		Close-mindedness	Managing committee	III
		Concurrence-seeking	Collaborators	III
		Open-mindedness	Lack of trust	III
			Ownership	III
		Innovativeness	Lack of conflict	III
	1, 3, 4	Trust	Family system	I
	1, 3, 5	Shared vision	Succession	III
		Trust	Board of directors	III
			Board of directors member	III
		Perceived conflict	Managers	III
			Teams	III
	1, 4, 5	Shared vision	Family meetings	I
		Benevolence	Innovativeness	III
		Integrity	Innovativeness	III
		Innovativeness	Trust	III
			Benevolence	III
			Integrity	III
	2, 3, 4	Ability	Non-family members	I
		Trust	Family business system	III
		Ability	Family business system	III
		Benevolence	Non-family members	III
			Family business	III
	2, 3, 5	Open-mindedness	Managers	III
			Collaborators	III
	2, 4, 5	Lack of shared vision	Innovativeness	I
		Innovativeness	Lack of shared vision	I
		Trust	Perceived conflict	III
		Perceived conflict	Trust	III
		Close-mindedness	Trust	III
	3, 4, 5	Shared vision	Board of directors	I
		Ability	Innovativeness	III
		Open-mindedness	Trust	III
		Innovativeness	Ability	III

**Table 7:** Summary of significant associative relationships.

**Dimension of analysis**						**Case/value of the radius (only values >1.96)**				
**Focal behavior**		**Conditional behavior**			**Quadrant**	**1**	**2**	**3**	**4**	**5**
**Shared vision**										
	Shared vision	Family Boundaries	Family system		III	7.39^**^	17.89^**^		3.59^**^	
			Family business system		I	23.72^**^	11.28^**^	5.39^**^		
			Non-family members		III	22.8^**^	3.92^**^	4.86^**^		
		Business roles	Board of directors' member^***^		III		5.32^**^		4.54^**^	5.55^**^
		Trust	Benevolence		III	6.12^**^	3.33^**^	2.61^**^	1.97^*^	4.82^**^
		Constructive conflict management	Perceived conflict		III	6.20^**^	3.87^**^	3.71^**^	5.36^**^	
			Multilevel system	Managing committee	I	18.69^**^	15.1^**^	10.31^**^		10.05^**^
				Ownership^***^	I	2.16^*^	12.88^**^			9.63^**^
				Family council	III	3.59^**^	4.26^**^			5.88^**^
				Family constitution	III	3.23^**^	5.38^**^			5.03^**^
				Family meetings	III	4.83^**^			5.3^**^	4.34^**^
				Board of directors^***^	I			3.76^**^	4.56^**^	10.16^**^
			Succession		III	8.93^**^	10.03^**^	2.49		5.19^**^
		Innovativeness	Innovativeness		I	10.88^**^	5.2^**^	5.6^**^		
			Risk-taking orientation	Risk-taking	I	13.97^**^	11.74^**^	7.09^**^		
				Risk-avoidance	I	10.06^**^	2.66	4.83^**^		
	Lack of shared vision	Family Boundaries	Family business system		I	3.43^**^	5.04^**^	3.25^**^		
			Non-family members		III	4.7^**^	2.21^*^	2.09		
		Trust	Lack of trust		I	8.45^**^	5.11^**^	6.07^**^		2.27^*^
		Constructive conflict management	Perceived conflict		I	10.78^**^	15.24^**^	15.17^**^		21.06^**^
		Innovativeness	Innovativeness		I		5.47^**^		2.21^*^	6.25^**^
			Risk-orientation	Risk-taking^***^	I	2.64^**^	3.99^**^			15.26^**^
**Trust**										
	Trust	Family Boundaries	Family business system		III		11.03^**^	12.27^**^	9.63^**^	
		Business roles	Board of directors' member		III	4.04^**^		3.62^**^		2.54^*^
			General manager		I	5.03^**^	2.5^*^	2.25^*^		9.46^**^
			Worker		III	4.99^**^	8.17^**^			12.05^**^
		Constructive conflict management	Perceived conflict		III		8.41^**^		3.73^**^	5.24^**^
			Succession		I	2.12^*^	4.64^**^		2.41^*^	
			Multilevel system	Collaborators	I	19.77^**^	2.04^*^	6.64^**^		5.9^**^
				Managing committee^***^	I	4.37^**^	17.8^**^			15.23^**^
				Board of directors	III	4.04^**^		3.79^**^		7.46^**^
		Innovativeness	Innovativeness		III	18.96^**^		2.18^*^	9.31^**^	14.05^**^
	Components									
	Ability	Family Boundaries	Family system		III	5.74^**^	4.17^**^		2.17^*^	
			Family business system		III		3.02^**^	10.47^**^	4.07^**^	
			Non-family members		I		7.2^**^	11.81^**^	5.26^**^	
			External environment		I	10^**^	5.63^**^	3.85^**^		
		Business roles	General manager		I	8.13^**^	2.96^**^			4.76^**^
			Workers		III	4.94^**^	2.66^**^			8.14^**^
		Constructive conflict management	Perceived conflict^***^		III	2.49^*^	4.69^**^		3.38^**^	3.55^**^
			Multilevel system	Family meetings	III	5.15^**^	2.35^*^		4.09^**^	2.07
				Ownership	III	2.63^**^	5.23^**^	2.83^**^		
				Family council	III	2.56^*^	5.27^**^		2.42^*^	
				Collaborators	I	4.19^**^	10.38^**^			3.24^**^
				Managing committee^***^	I	4.82^**^	13.44^**^	2.2^*^		2.77^**^
				Family constitution	III	2.42^*^	2.31^*^			2.78^**^
		Innovativeness	Innovativeness^***^		III			2.52^*^	3.93^*^	6.37^**^
			Collaborative innovation		I	7.8^**^	6.3^**^			6.7^**^
	Integrity	Innovativeness	Innovativeness^***^		III	9.64^**^			2.74^**^	2.49^*^
	Benevolence	Boundaries	Family system		I	6.53^**^	4.65^**^		16.55^**^	
			Family business system		III		8.06^**^	3.23^**^	4.39^**^	
			Non-family members		III		2.48^*^	3.22^**^	2.41^*^	
		Constructive conflict management	Multilevel system	Board of directors	III	2.4^*^	4.39^**^	3.39^**^	9.87^**^	6.06^**^
				Teams	III	3.67^**^	2.1^*^			2.83^**^
		Innovation	Innovativeness		III	15.88^**^			3.52^**^	6.88^**^
	Lack of trust	Constructive conflict management	Multilevel system	Board of directors	I	4.24^**^	11.52^**^		2.34^*^	3.04^**^
			Processes	Close-mindedness	I	9.89^**^	2.07^*^			2.15^*^
**Constructive conflict management**										
**Perceived conflict**	Perceived conflict	Boundaries		External environment	III	8.72^**^	12.81^**^	3.1^**^		
		Business role		Managers	III	17.33^**^		2.62^**^		2.54^**^
			Trust		III		8.41^**^	3.73^**^	5.24^**^
		Trust		Ability	III	2.49^*^	4.69^**^		3.38^**^	3.55^**^
		Multilevel system		Family constitution	I	10.42^**^	5.5^**^			2.7^**^
			Teams		III	11.41^**^	4.09^**^		3.61^**^
		Innovativeness		Innovativeness^***^	III	15.47^**^	7.81^**^	9.65^**^		
	Lack of conflict	Multilevel system		Managing committee	III	4.38^**^	2.89^**^			2.42^*^
				Collaborators	III	6.62^**^	2.41^*^			2.83^**^
		Innovativeness		Innovativeness	III	7.23^**^	2.84^**^			3.95^**^
Types										
	Task conflict	Business role		General manager	I		11.67^**^	4.63^**^		3.36^**^
	Work-family conflict	Family history			I	10.29^**^	2.81^**^	7.95^**^	
	Succession	Trust			I	2.12^*^	4.64^**^	2.41^*^		
Processes	Conditional behaviors							
	Open-mindedness	Boundaries		External environment	III	8.88^**^	3.54^**^	3.07^**^		
		Business role		Managers^***^	III		3.41^**^	4.7^**^		4.58^**^
		Trust	Trust		III			5.97^**^	5.62^**^	3.81^**^
			Lack of trust		III	5.5^**^	2.47^**^		2.93^**^	
		Multilevel system		Family meetings	I	2.94^**^	4.13^**^		5.25^**^	
				Family council	I	7.68^**^	6.68^**^		2.73^**^	
				Ownership	III	3.01^**^	3.03^**^			6.87^**^
				Collaborators	III	33.04^**^	5.5^**^	2.79^**^		2.03^*^
	Close-mindedness	Trust	Trust		III		4.46^**^		2.03^**^	2.26^**^
			Lack of trust		I	9.89^**^	2.07^*^			2.15^*^
		Multilevel system		Managing committee	III	2.49^*^	3.53^**^			2.8^**^
	Concurrence-seeking	Multilevel system		Ownership	I	4.41^**^	7.74^**^			2.11^*^
				Family meetings	I	14.17^**^	18.08^**^		4.14^**^	
				Collaborators	III	4.83^**^	4.77^**^			3.33^**^
Innovativeness	Innovativeness	Shared vision			I	10.88^**^	5.2^**^	5.6^**^		
		Lack of shared vision^***^			I		5.47^**^		2.21^*^	6.25^**^
		Trust		Trust	III	18.96^**^			9.31^**^	14.05^**^
			Lack of trust		III	18.17^**^	3.41^**^		3.06^**^	3.78^**^
			Components	Benevolence	III	15.88^**^			3.52^**^	6.88^**^
				Integrity^***^	III	9.64^**^			2.74^**^	2.49^*^
				Ability	III			2.52^*^	3.93^**^	6.37^**^
		Constructive conflict management	Perceived conflict^***^		III	15.47^**^	7.81^**^	9.65^**^		
			Lack of conflict		III	7.23^**^	2.84^**^			3.95^**^
			Succession		III	21.36^**^	17.1^**^	5.7^**^	3.65^**^	9.69^**^
	Collaborative innovation	Trust	Components	Ability	I	7.8^**^	6.23^**^			6.7^**^

* *Significant associative relationship (*p*-value <0.05)*.

** *Very significant associative relationship (*p*-value <0.01)*.

*** *Statistically significant associative relationship presented in five cases although in different quadrant to the existent across at least three cases*.

As it is shown in Tables 6, 7, the parallel significant results across at least three cases were concentrated in two opposing quadrants (I and III) expressing the existence of two types of associative relationships between focal and conditional behaviors: mutual activation and mutual inhibition (see Table 7). The presence of significant associative relationships was ample (Total = 99) given the wide range of focal behaviors and conditional behaviors selected for analysis. The presence of three associative relationships that were consistent across five cases was significant. These three relationships indicated that shared vision, benevolence (specific component of trust) and innovativeness did not emerge in presence of narratives concerning benevolence, board of directors and succession, respectively.

It was also noticeable that 11 associative relationships were coinciding across three combinations of four cases. Cases 1, 2, and 5 showed the higher level of similarity given the existence of the higher number of coinciding associative relationships (Total = 27).

In the following sub-sections, we will present the results of polar coordinate analysis differentiating the focal behaviors and conditional behaviors which were selected for analysis according to our previous theorization.

### Looking Into Family Boundaries

The four subdimensions of family boundaries (family system, family business system, non-family members, and external environment) selected as conditional behaviors showed significant associative relationships with shared vision and trust. In this sense, we found that the presence of an inspiring vision for the future or shared vision was activated in presence of narratives concerning family business systems whereas narratives regarding family members and non-family members were inhibitors of narratives of shared vision. The perception of a lack of shared vision and family business system were mutually activated, whereas lack of shared vision and non-family members were mutually suppressed or inhibited.

Concerning trust, the family boundaries indicated that narratives regarding the several subsystems activated or inhibited narratives depending if these referred to the perception of trust, lack of trust or the specific components of trust. We found that mentions to the perception of trust did probably not emerge in the presence of narratives regarding the family business system. Indeed, references to a family system and family business system showed this inhibitory role of perception of the component of ability whereas elusions to non-family members and the external environment showed an activation effect of the perception of the capacity or ability to manage the tasks as a trait of trust. For the component of benevolence, we found the opposite direction such that elusions to the family system activated the perception of caring or the affection-based component of trust (benevolence) whereas family business system and non-family members exerted an inhibitory role.

Narratives regarding the external environment and perceived conflict were mutually inhibited. The same type of relationship (mutual inhibition) was found between those narratives referred to the external environment and open-mindedness. The external environment seems to be perceived as trustful as long as narratives of ability and external environment are mutually activated.

### Relationships Between Shared Vision and Trust

**Figure 3:**
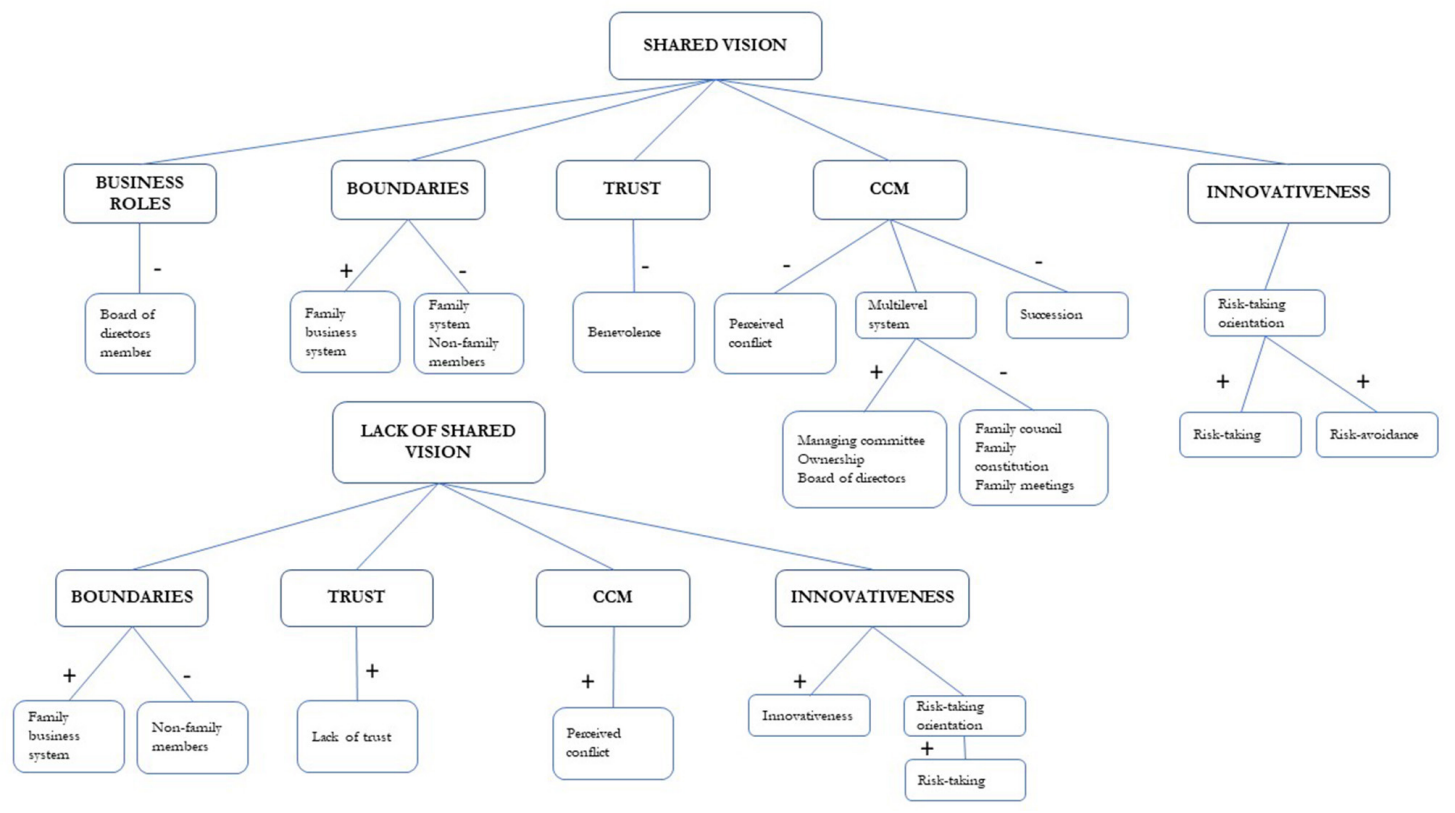
Structure of significant associative relationships of shared vision dimension (+ Quadrant I/– Quadrant III).

**Figure 4:**
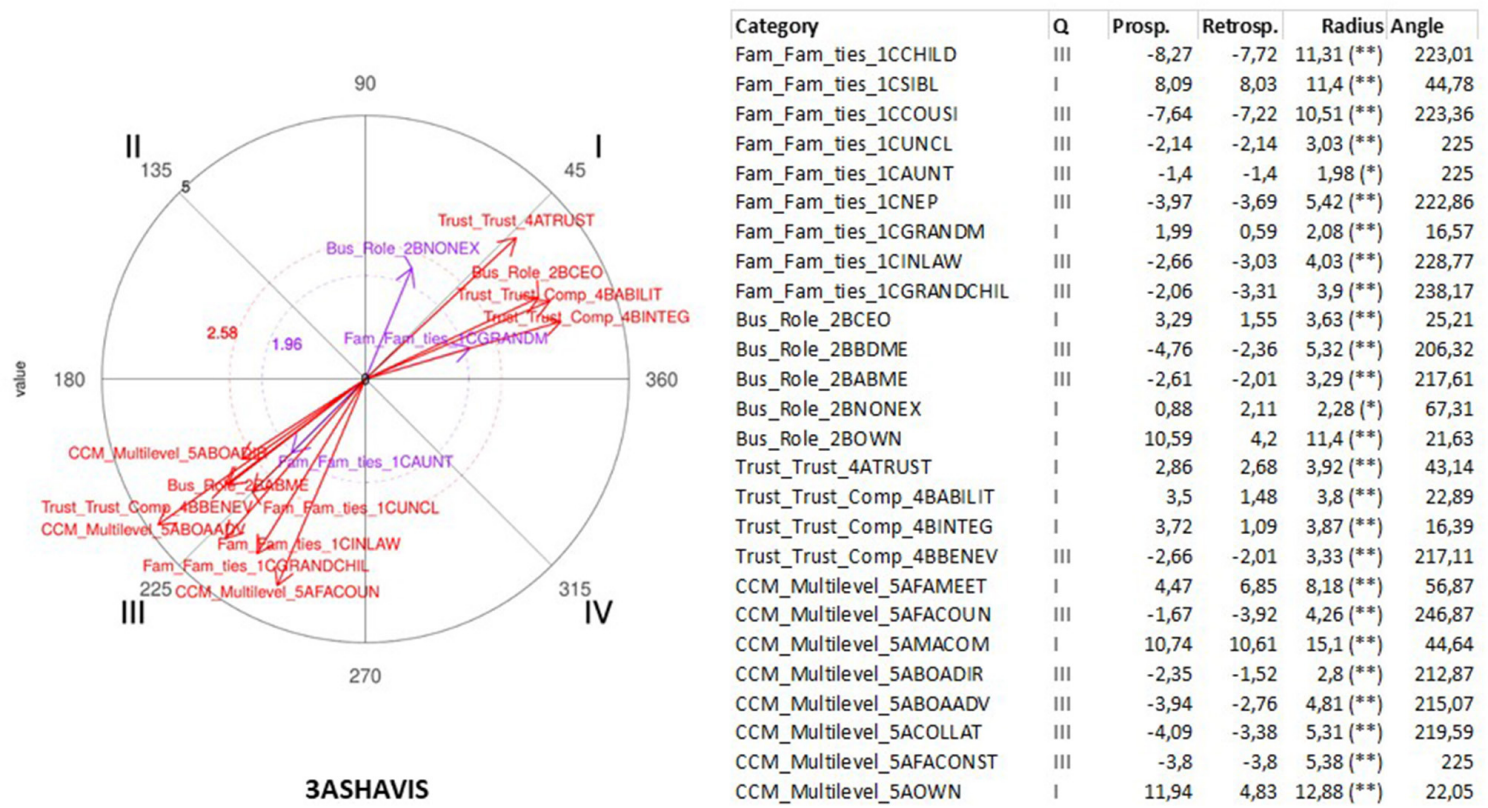
Example A of vectorial map of the statistically significant relationships. The cart shows the vectorial map of the statistically significant relationships for the category of shared vision (3ASHAVIS) considering as focal behavior, and family boundaries (1A), generation (1B), family ties (1C), business roles (2B), trust (4A), components of trust (4B), lack of trust (4C), multilevel system (5A), perceived conflict (5B), types of conflict (5C), processes (5D), third-party assistance (5E), succession (5F), innovativeness (6A), risk-taking orientation (6B), and decision-making pace (6D), as conditional behaviors. At the table the results of the polar coordinate analysis are presented. The significance level was fixed at *p < 0.05 and **p < 0.01.

As shown in Figure 3, shared vision showed a rich structure of associative relationships with family and business dimensions, trust, constructive conflict management, and innovativeness. In this regard, we found evidence about the relationship between shared vision and trust. First, we found that shared vision showed an associative relationship of mutual inhibition with the affective component of trust or benevolence. Second, the perception of lack of shared vision was followed by the perception of lack of trust which is consistent with our theorization about the mutual influence between both dimensions (For an example of a vectorial map of shared vision as focal behavior, see Figure 4).

### Relationships Between Shared Vision and Constructive Conflict Management

The indirect observation reported that shared vision narratives were inhibited in presence of mentions to controversies that produced tensions or interference (perceived conflict). Something similar was found between the presence of narratives of shared vision and references to conflict related to the process of transference of power, managerial roles and ownership (succession) in which they were mutually inhibited. We indirectly observed that shared vision was activated in the presence of mentions to the managing committee, ownership (this associative relationship was observed across the five cases although in two of them it was in a different quadrant), and board of directors (it was coinciding in the five cases but in two of the cases it was in a different quadrant). On the contrary, mentions to family meetings, family council and family constitution inhibited the emergence of narratives regarding future goals or shared vision. Relationships between shared vision and processes of constructive conflict management are not supported by consistent findings across at least three cases. Although we found that a perception of lack of shared vision and perceived conflict exhibited a mutual activation relationship.

### Relationships Between Shared Vision and Innovativeness

Findings supported the relationship between shared vision and mentions to the tendency of the firm to engage and support processes oriented to result in new products, services or processes (innovativeness) in the expected direction given that narratives of future goals or shared vision are activated in the presence of these mentions to innovativeness. The same relationship was found with the subdimension of risk-taking orientation (both categories of risk-taking and risk-avoidance). Narratives of lack of shared vision also showed this relationship of activation with references to innovativeness and risk-taking orientation.

### Relationships Between Trust and Constructive Conflict Management

**Figure 5:**
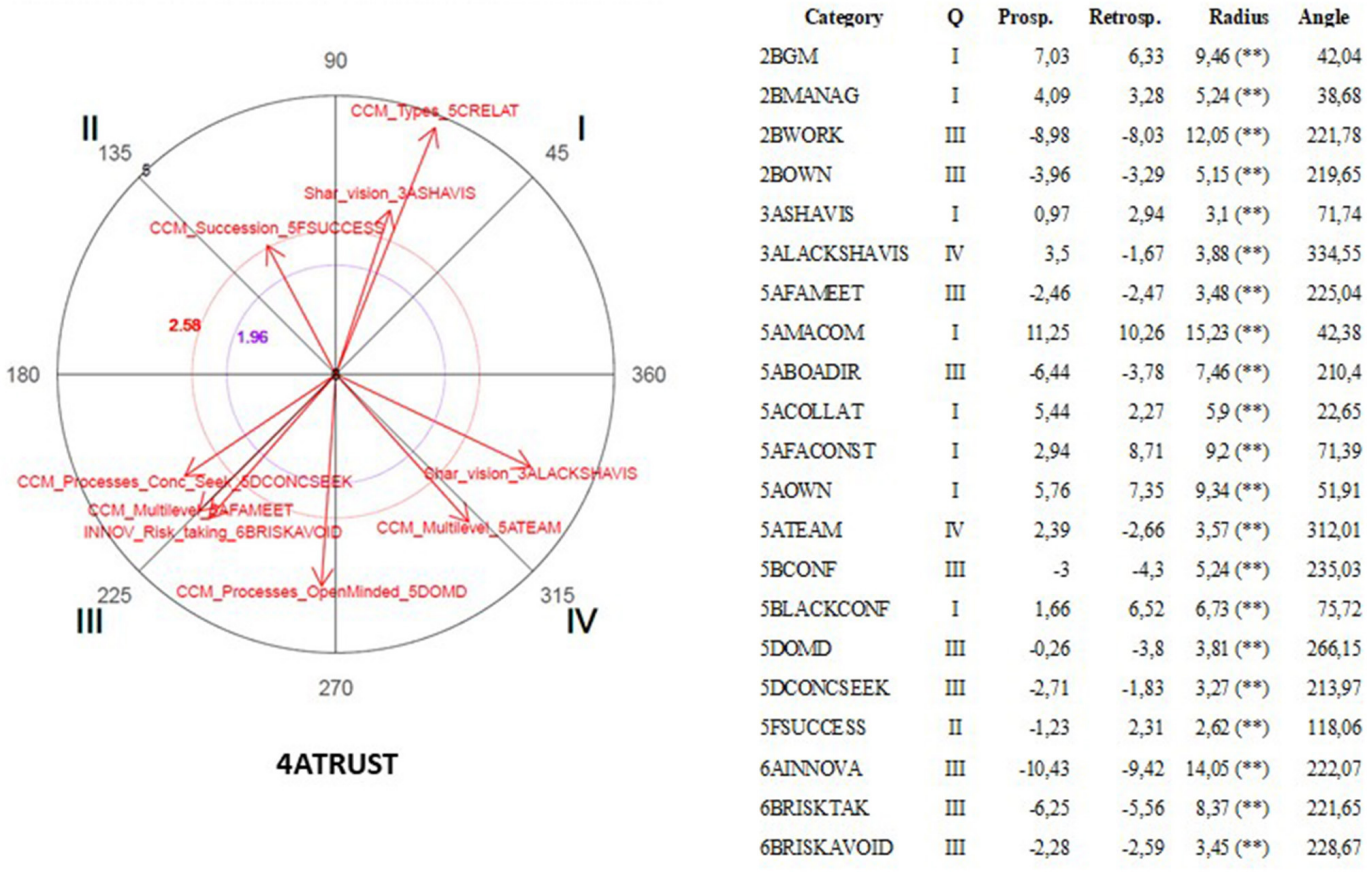
Example B of vectorial map of the statistically significant relationships. The graph represents the vectorial map of the statistically significant relationships for the subdimension of trust (4ATRUST) considering as conditional behaviors the following: family boundaries (1A), generation (1B), family ties (1C), family history (1D), business roles (2B), shared vision (3), multilevel system (5A), perceived conflict (5B), types of conflict (5C), processes (5D), third-party assistance (5E), succession (5F), innovativeness (6A), and risk-taking orientation (6B). The table contains the results of the polar coordinate analysis. The significance level was fixed at **p < 0.01.

Narratives regarding the perception of trust inhibited narratives of perceived conflict whereas narratives of trust were activated in presence of narratives of a specific conflict of succession. We found evidence about the relationship between narratives of trust and narratives regarding the different levels at the organization where conflict management took place. In this sense, trust was indirectly observed alongside mentions to collaborators and managing committee whereas mentions of trust were inhibited in presence of narratives concerning the board of directors. It is noticeable that an associative relationship between trust and managing committee was coinciding across five cases although not in the same quadrant. In Figure 5, it is shown an example of a vectorial map obtained from the polar coordinate analysis considering trust as a focal behavior.

From the scrutiny of the specific components of trust, we found a relationship between mentions to ability and perceived conflict. They were mutually inhibited. We also found that mentions to ability were activated in presence of elusions regarding the levels of collaborators and managing committee and that they were inhibited when participants referred to family meetings, family council, family constitution and ownership. Concerning narratives regarding the affective component of trust or benevolence, they were inhibited in presence of mentions to the board of directors and teams. We also found that narratives of lack of trust and trust were related to open-mindedness where they exerted an inhibitory role. Whereas, in close-mindedness narratives of trust exerted an inhibitory role and lack of trust activated the references to close-mindedness.

### Relationships Between Trust and Innovativeness

We found that narratives of trust and innovativeness were related. Perception of trust and their components of benevolence, ability and integrity were associated with innovativeness in the sense that they were mutually inhibited. However, the perception of ability which was activated in the presence of narratives related to engaging innovation in collaboration with external parties or collaborative innovation.

### Examining Constructive Conflict Management

**Figure 6:**
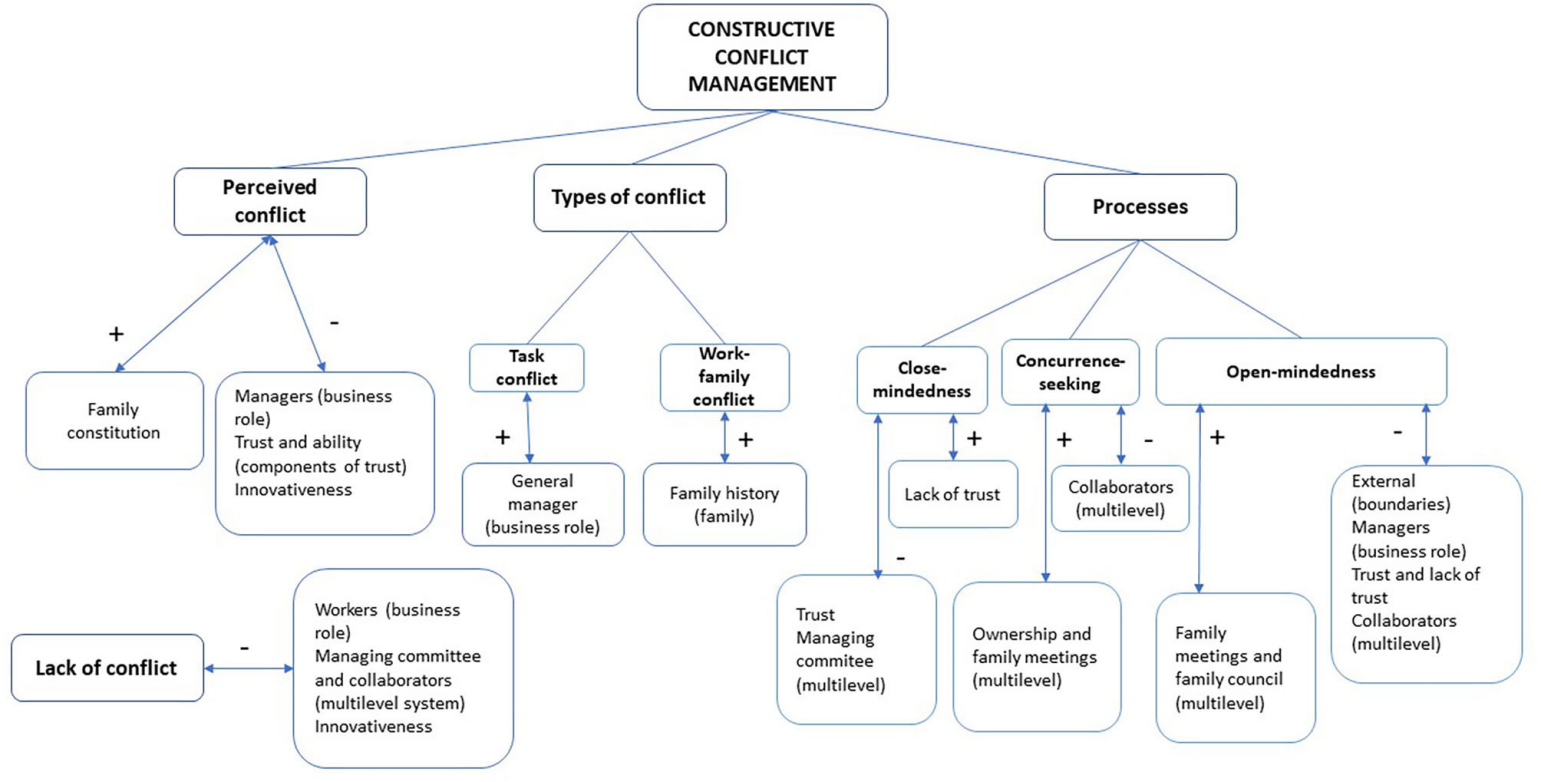
Structure of significant associative relationships of constructive conflict management dimension (+ Quadrant I/ Quadrant III).

Given that we were interested in understanding constructive conflict management we conducted several analyses considering the different subdimensions as focal behaviors. As it is shown in Figure 6, we found evidence about the presence of narratives regarding conflict management at different levels of the organization such that the perception of conflict was activated alongside references to family constitution and inhibited when participants talked about teams. Another finding was related to the relationship between mentions to work-family conflict and family history such that where they were mutually activated.

We found evidence about the presence of open-mindedness at different levels of the organization. Precisely, findings indicated that this process is more probably present in family meetings and family council whereas it was not referred to during the elusions of ownership and collaborators. Concurrence-seeking and open-mindedness shared in some sense this structure of relationships, such as that narratives regarding concurrence-seeking and family meetings and ownership were mutually activated though they were inhibited when participants referred to collaborators. In managing committees, it close-mindedness did not seem present as the inhibitory relationship between narratives of both types indicated.

### Relationships Between Constructive Conflict Management and Innovativeness

**Figure 7:**
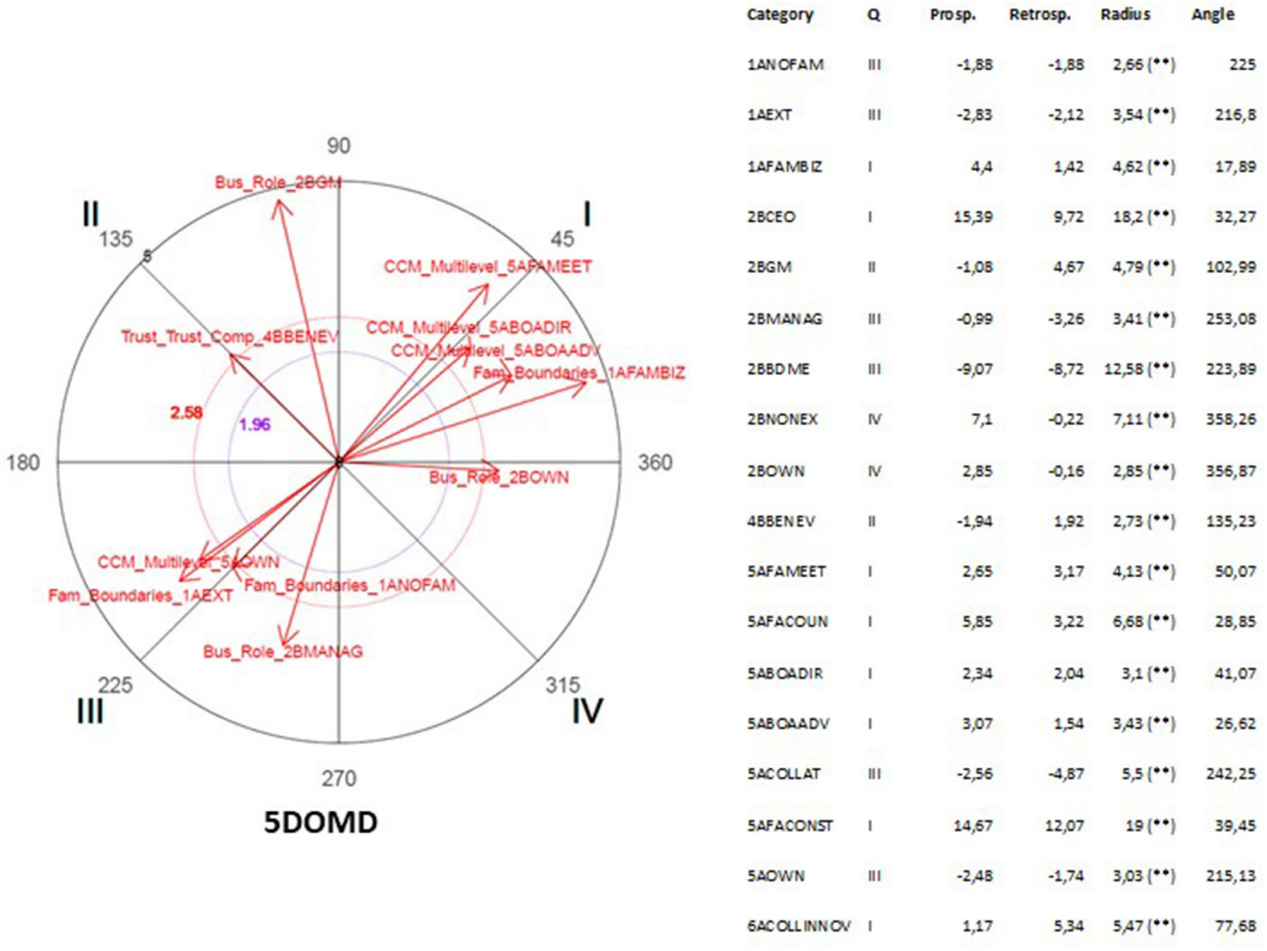
Example C of vectorial map of the statistically significant relationships. The graph exhibits the vectorial map of the statistically significant relationships for the subdimension of open-mindedness (5DOMD) considering the following as conditional behaviors: family boundaries (1A), business roles (2B), trust (4A), trust components (4B), lack of trust (4C), multilevel system (5A), and innovativeness (6A). The table contains the results of the polar coordinate analysis. The significance level was fixed at **p < 0.01.

As it is shown in Figure 7, innovativeness and conflict were associated such that they mutually inhibited. When participants narrated experiences of innovativeness in their companies they did not talk about perceived conflict, lack of conflict or succession.

### Business Roles Matter

Concerning the several business roles played in the company, the results obtained indicated that they conditioned shared vision, trust, constructive conflict management and innovativeness in different senses.

For instance, we found that references to the members of the board of directors suppressed the presence of narratives of shared vision and trust. In other words, if the participants narrated experiences regarding the board of directors, these were not followed by mentions to shared vision and trust. It is noticeable that mentions to the general manager activated narratives concerning the component of trust related to personal skill and capacities (ability) whereas references to workers exerted the opposite effect, it meant that if the participants referred to ability they did not refer to trust stemmed on ability. Findings supported that perceived conflict and open-mindedness were not referred to managers.

## Discussion

In this research, we provide evidence about the use of indirect observation as a rigorous approach to study narratives and extracting conclusions about complex phenomena (Anguera et al., 2018). In addition, the combined use of mixed methods and multiple case has demonstrated being a suitable design to use in studies in the context of family firms. It offers interesting opportunities to quantify the existent relationships between the different dimensions of a complex reality and find shared structures of significant associative relationships between the cases allowing to account for the existence of a multiple case (Anguera, 2020).

Therefore, this study yields empirical evidence about the unique set of cognitive, emotional, and behavioral responses involved in conflict situations (García et al., 2016; Alvarado-Alvarez et al., 2020). The general picture emerging from the results indicates that trust and shared vision play a relevant role in the constructive conflict management of family firms, and they are deeply ingrained as elements of constructive conflict management. This research also sheds light about the possible influence of social processes, such as shared vision, trust, and conflict in innovativeness.

Our findings confirm previous theoretical work, empirically showing that shared vision and trust are cognitive and relational roots of constructive conflict management (cf. Alvarado-Alvarez et al., 2020). Results suggest that shared vision and trust have their path (cognitive and emotional) to influence constructive conflict management and innovation in family firms. Looking into the results, we find that shared vision and trust are related but seem connected by the cognitive component of trust (ability) and not through affective elements of trust (e.g., benevolence).

Indeed, affection-based trust (benevolence) would hinder the perception of future goals or shared vision. It seems that benevolence stems from family ties and shared vision is referred to business goals according to the results. This finding might explain that shared vision does not seem to be a subject of conversation in family governance forums (e.g. family meetings, family council). A plausible explanation for this, is that shared vision is an antecedent of the creation of a governance bodies, particularly in siblings partnership stage, therefore shared vision is not a topic of conversation in family governance forums. In fact prior research suggests that the more aligned the values and vision of the family, the more they develop governance structures (Parada, 2015). This finding is also consistent with the practices of governance of the cases studied which distinguishes spaces for debating family issues (e.g., family council) and business strategy (e.g., board of directors or advisory board). Our findings suggest that shared vision is more focused on business goals than on family goals. From the findings, it is not possible to conclude that shared vision comprises non-family related goals. Previous studies have referred to the salient role of non-family related goals of families in business (Berrone et al., 2012; Kotlar and De Massis, 2013).

In this sense, our results contradict our expectations about shared vision triggering bonding and emotional contagion, as seen in previous studies (c.f. Boyatzis et al., 2015; Wang and Shi, 2020). However, it supports the argument that shared vision is different from cohesiveness (around family ties *per se*) which would lead to groupthink (Kidwell et al., 2012; Lord, 2015).

Our analyses indicate that when families in business are in the middle of conflicts related to succession for instance, shared vision does not easily emerge. Apparently, this finding contradicts current evidence about shared vision having a positive influence on succession (e.g., Overbeke et al., 2015; Daspit et al., 2016). However, it may suggest that during a succession process the incumbent and the successor need to negotiate their goals and future expectations to succeed during this stage (Caputo and Zarone, 2019).

Data provides evidence about the importance of context for the emergence of trust (Kramer, 1999). For instance, benevolence is perceived as related to family relationships (Steier, 2001; Eddleston et al., 2010), whereas the perception of ability is related to managerial roles and decision-making at the business level (Mayer et al., 1995). The presence of altruism may explain to some extent that narratives regarding skills and competences as a source of trust or ability were not followed by references to family meetings, family council or the ownership level (Lubatkin et al., 2005; Kidwell et al., 2018). This study depicts the different sides of trust in family firms (Eddleston and Morgan, 2014).

In this study, we may confirm the existence of specific components of trust (ability, benevolence, and integrity) (Schoorman et al., 2007) that have different effects on constructive conflict management in family firms. For instance, shared vision is related to the cognitive component of trust (ability) with specific implications in constructive conflict management at the organizational level where it takes place. Conversations about future projects and goals are more proper for business levels of governance. However, we do not find evidence about these debates being open-minded. We find that open-mindedness is associated to spaces of conversations led by family members, such as family meetings and family council, confirming the conceptualization of family ties fertilizing the ground for the emergence of constructive conflict management (Alvarado-Alvarez et al., 2020). The prevalence of open-minded debates and concurrence-seeking in family forums suggest the need to constructively manage their conflicts to maintain trust and family harmony (Kidwell et al., 2012).

An interesting insight we can extract from this study is that the agile and frequent communication may propitiate a better communication which prevents destructive conflict dynamics. Moreover, the presence of concurrence-seeking at the ownership level underlines the necessity of reaching agreements or consensus (e.g., shareholders policies; Ensley and Pearson, 2005). We can also observe the pernicious effect of a lack of trust hindering open-mindedness or favoring close-mindedness.

Moreover, results suggest that collaboration between family and non-family members may be hampered by a lack of shared vision (Waldkirch, 2020). Indeed, findings suggest that the board of directors is not perceived as a place where trust flourishes which can be considered as harmful for the social dynamics and the decision-making of this forum (Eddleston et al., 2010) and psychological capital within the members of the organization (León-Perez et al., 2016; Meier and Schier, 2016; Tang, 2020).

A finding which requires further exploration is that close-mindedness and trust are reciprocally influenced. It is suggesting that certain topics may not be openly discussed or that some barriers exist to effectively deal with conflict (Kiernan et al., 2019). Perhaps the perception of lack of trust between family and non-family members is a plausible explanation which merits further studies.

Despite these interesting insights, we consider that the role of trust in open-mindedness requires more scrutiny. The results indicate that open-mindedness is inhibited by the perception of trust and lack of trust. It may reflect some differences between the cases but it can also be an expression of the different roles of trust as an antecedent and outcome of constructive conflict management. If parties trust each other, they do not perceive the need of managing conflict because they may address it through communication, and consequently, they would not associate trust and open-mindedness. Conversely, if the parties lack trust, they do not feel safe to openly talk about conflict (Deutsch, 2011).

Furthermore, our results are tightly connected to boundaries theory (Sundaramurthy and Kreiner, 2008; Knapp et al., 2013) and systems theory (Distelberg and Sorenson, 2009) because participants differentiate between family, family business as a whole entity, non-family and external environment. These boundaries have several implications in terms of resources and conflict management. From the family system, benevolence can be considered a relational resource (Pearson et al., 2008). The presence of benevolence can be an outcome of behavioral integration and relational governance implemented by the companies studied (Rosenkranz and Wulf, 2019) through family governance tools (e.g., family council, family meetings). Whereas, perceiving ability in the external environment (e.g., competitors, professional associations, entrepreneurial ecosystem) contributes to the collaborative innovativeness engaged by the cases studied (Feranita et al., 2017).

The non-family members subsystem and external environment are perceived as richer in skills and capacities for good performance (Bettinelli, 2011; Tabor et al., 2018). Family firms rely on high levels of trust for the creation of knowledge with allies (Feranita et al., 2017; Bouncken et al., 2020). In general terms, non-family members seem to have dissimilar levels of shared vision and trust (Tabor et al., 2018). Some explanations may be related to the existence of different goals (Jaskiewicz and Luchak, 2013), family bias (Tabor et al., 2018), and social categorization (Kramer, 1999; Waldkirch et al., 2018). The relationship between task conflict and general manager narratives observed in this study support evidence about the existence of this type of conflict at managerial levels and its implications in terms of stress and psychological well-being (Guerra et al., 2005; De Clercq and Belausteguigoitia, 2017).

The differentiation between different levels of constructive conflict management and their implications in terms of shared vision and trust may provide evidence about the influence of family business advising in developing governance practices (Strike et al., 2018). The fact that benevolence is not perceived at the business level of conflict management is an intriguing finding. Further exploration may be required to understand the implications of emotions and trust in family business governance (Eddleston et al., 2010; Kellermanns et al., 2014).

An interesting side result is that conflict and family constitution mutually reinforce each other. It is consistent with current evidence about some governance tools, such as Family Constitutions, or Family Protocols that are used to manage conflict in family firms (or conflict triggers their implementation) (Arteaga and Menéndez-Requejo, 2017).

Our study brings evidence that shared vision would promote innovativeness which contradicts some theorization about the potential harming of a shared culture as a sort of “collective blindness” that may inhibit the pioneering process and consequently innovation (Carnes and Ireland, 2013). Our findings suggest that the diverse approaches used to allocating resources (risk orientation) require to undertake conflict management processes at different levels of the organization (Borbély and Caputo, 2017; e.g., managing committee and board of directors). At the same time, shared vision is a driver to achieve innovativeness (Neff, 2015) and represents a collective cognition between family and non-family members (Madison et al., 2020).

The processes of constructive conflict as open-mindedness or concurrence-seeking does not emerge with the same strength in the picture of innovativeness. Although the findings suggest that the perception of conflict or the lack of conflict may hamper innovativeness, which is consistent with the paradoxes of conflict and innovation described in scientific literature: too much conflict hampers innovation but at the same time specific levels of conflict trigger organizational innovation (Vollmer, 2015). Something that emerges from the analysis is the importance of working on diminishing the perception of lack of shared vision to positively impact on innovativeness (Neff, 2015).

### Contributions

This research reports interesting insights about trust and constructive management in the unique context of family firms (Elgoibar et al., 2016). Therefore, it extends current knowledge because it brings empirical evidence about the specific role of shared vision and trust in constructive conflict management and innovativeness in family firms (Alvarado-Alvarez et al., 2020). It also describes the dynamic character of constructive conflict as an emergent process in family firms (Kozlowski and Chao, 2012). This research refines our understanding of the role of cognitions in the conflict management of family firms following the current stream of research in family business (Kammerlander and Breugst, 2019). This study also depicts the importance of family governance practices for managing conflict in family firms contributing to understand their uniqueness in terms of constructive conflict management (Alvarado-Alvarez et al., 2020). Methodologically, it represents an evidence of the adequacy of a mixed method approach to study complex realities (e.g., family firms; Plano Clark et al., 2008). Indeed, it represents a novel methodological approach based on systematic observation, specifically on indirect observation (Anguera et al., 2017, 2020), which may report an excellent advance for both fields of conflict management and family business research.

### Practical Implications

Family governance is a fundamental level of constructive conflict management in family firms (Berent-Braun and Uhlaner, 2012; Suess-Reyes, 2017) as we confirm in this research. Families address critical conversations in spaces as informal gatherings and family councils demonstrating that institutions and relationships contribute to trust (Lewicki, 2006). In this study, the importance of managing conflicts of succession emerges as a relevant finding which opens interesting avenues for mediators, conflict managers and organizational psychologists (e.g., Haynes et al., 1997). We encourage the development of education programs adapted for families in business and the advisory practices from a psychological approach which encourage the adoption of constructive conflict management.

Trust promotion between family and non-family members should be included in the agenda of family firms to boost collaboration and innovativeness through constructive conflict management (Bennedsen and Foss, 2015). Indeed, the use of open-mindedness may help to deal with the existence of task conflict and cognitive conflict at a business level.

### Limitations and Further Research Agenda

Despite the strengths of this research, we acknowledge that it is not exempt from limitations. We are aware that this study is an initial step to understand the dynamics of constructive conflict in family firms. It opens interesting avenues to further studies. For instance, the exploration of the relationships between shared vision, bonding and relational climate in the family firm require further exploration. Further research may explore a more heterogeneous group of cases in terms of their levels of innovativeness, size or geographical situation. Moreover, studying cases in different generational stages of ownership is a good avenue to extend this study. Indeed, studying cases which might not have been assisted by family business advisors may be an interesting opportunity. In this study, narratives provided by family members are more prevalent. Further studies may explore in-depth the perspective from non-family members.

Further studies may investigate the potential of conflict management as a tool for balancing work and family in the context of family firms (Lu et al., 2012). Indeed, the exploration at the individual level of conflict management in family firms as a relevant contributor to personal well-being is a promising area (León-Perez et al., 2016). Further inquiries are needed about the role of third parties in conflict management of family firms and their contribution to trust promotion practices (Strike et al., 2018). Exploring their vision of conflict and open-mindedness merits attention.

Given that conflict is in the area of sensitive issues (Jehn and Jonsen, 2010), the use of a systematic direct observation (Sánchez-Algarra and Anguera, 2013; Anguera et al., 2017, 2020) of real-life situations in family businesses (e.g., family councils, meetings of the board of directors, informal family meetings) and evaluation program designs may be alternative ways to approach to the phenomena (Chacón-Moscoso et al., 2013; Portell et al., 2015). Further studies which explore the possibilities that may offer constructive conflict management to family firms in current trends as digital transformation are encouraged (Vollmer, 2015; Vaska et al., 2020).

## Conclusions

This research sheds light on the uniqueness of constructive conflict management in family firms. We provided empirical evidence of shared vision and trust as roots of constructive conflict management observed at different levels of the organization. Trust management demonstrates critical importance for obtaining constructive outcomes of conflict in this type of organizations. Conflicts of succession emerges as a critical moment for developing trust and shared vision. Although the role of conflict in innovativeness is confirmed, it is necessary to further explore in-depth open-mindedness and concurrence-seeking in family firms. This study paves the way to further research, which looks into family firms from a psychological perspective.

## Data Availability Statement

The raw data supporting the conclusions of this article will be made available by the authors, without undue reservation.

## Ethics Statement

The study has been carried out following the ethical guidelines and procedures of the Doctorate Program of Psychology of Communication and Change of the both Autonomous University of Barcelona and University of Barcelona, to which the first author is a Doctoral candidate and the ethical standards for research established by the American Psychological Association (2017). All participants gave their informed consent to participate in the research in conformity with the Declaration of Helsinki before making the audio recording and data collection. Personal information of participants was replaced and not provided to the coders of audio recordings and transcripts to ensure confidentiality.

## Author Contributions

CA-A carried out the data collection, literature review, research design, writing the manuscript, coded the textual material, and trained and supervised the coders who participated in the data quality control procedures. IA and MP made a substantial, direct and intellectual contribution through supervising all the stages of the research process, and reviewing the several drafts of the article. MA supervised the method and conducted the polar coordinate analysis. All authors contributed to the article and approved the submitted version.

## Conflict of Interest

The authors declare that the research was conducted in the absence of any commercial or financial relationships that could be construed as a potential conflict of interest. The handling editor declared a shared affiliation with several of the authors CA-A, IA, and MA at time of review.
