# Supplementary material for: Unraveling the Role of Shared Vision and Trust in Constructive Conflict Management of Family Firms. An Empirical Study From a Mixed Methods Approach
Source: Front Psychol. 2021 Jun 15;12:629730. doi: 10.3389/fpsyg.2021.629730 (PMC8239237; doi:10.3389/fpsyg.2021.629730)
Supplement: Supplementary file 1 [file Data_Sheet_1.docx]

**Questions guide for interview**

Although the interviews were open-ended, the interviewer considered the following questions as a reference.

*Questions addressed to family members*

1. The general context of the family business

a. Tell me about your history in the family business.

b. How do you describe your family business?

c. Explain some situation which might illustrate how are the relationships between family members inside the business.

d. Give some examples of the role played by the family in the business and how business affects the family.

e. How do you describe the goals of this family in the business?

2. Constructive conflict management.

a. Tell me about some situation where family coped with a conflict in the business.

b. What were the main effects of that conflict both in family and business?

c. Tell me about a recent situation where a family member would have disagreed about business issues. How did you deal with these differences and controversies?

d. Tell me about some situation that might be an example of how family members make decisions in the company.

e. Tell me about some conflict related to decision making in the company. How did you manage it?

3. Innovation

a. Explain how the innovation of your family business is.

b. Give some examples of the recent innovation developed in the business.

c. Tell me about how innovation is taking place.

*Questions addressed to non-family managers*

1. Talk to me about your history in the family business.

2. Describe the last innovation projects that you are undertaking in your area.

3. How these initiatives are taking place. What resources and obstacles have you found.

4. How is the innovation process deployed regarding change management and decision-making?

5. What are the plans for innovation?

6. In your opinion, what role does the family play in the innovation of the business?
