# Supplementary material for: Unraveling the Role of Shared Vision and Trust in Constructive Conflict Management of Family Firms. An Empirical Study From a Mixed Methods Approach
Source: Front Psychol. 2021 Jun 15;12:629730. doi: 10.3389/fpsyg.2021.629730 (PMC8239237; doi:10.3389/fpsyg.2021.629730)
Supplement: Supplementary file 2 [file Data_Sheet_2.docx]

| **Dimension** | **Subdimension** | **Categories** | **Code** | **Description** |
| --- | --- | --- | --- | --- |
| **Family 1** | Boundaries  A | Family  Non-family  Family business  External environment | **1AFAM**  **1ANOFAM**  **1AFAMBIZ**  **1AEXT** | Narratives are referring to the members of the controlling owner family or not, delimitating clear boundaries between family and non-family members of the company (Knapp et al., 2013), the family firm as a whole and the external context of the family firm (e.g., competitors or other family firms) (Cruz & Nordqvist, 2012). |
|  | Generation  B | Founder  Second generation  Third generation  Fourth generation | **1BFOUND**  **1B2G**  **1B3G**  **1B4G** | Awareness that different generations involved in the family business exist. The reference point used to be the founder (s) considered the first-generation (Cruz & Nordqvist, 2012; Kraiczy et al., 2012; Lansberg, 1999). |
|  | Family ties  C | Spousal  Parent-children  Sibling  Cousin  Uncle, aunt  Niece, nephew  Grand-father  Grand-mother  Grand-children  In-law | **1CSPOU**  **1CCHILD**  **1CSIBL**  **1CCOUSI**  **1CUNCL**  **1CAUNT**  **1CNIEC**  **1CNEP**  **1CGRANDF**  **1CGRANDM**  **1CGRANDCHIL**  **1CINLAW** | The different family kinship (Ensley & Pearson, 2005; Pearson et al., 2008) referred to in the narrative. Also considered as the salient family subsystem in which the informant belongs, such as the sibling or the cousin group. |
|  | Family history  D | Family history | **1DFAMHIST** | Narratives related to the family's historical milestones or their individuals typically are connected to the business history (Suddaby & Jaskiewicz, 2020). |
| **Business 2** | Characteristics  A | Size  Industry  Market | **2ASIZE**  **2AINDUST**  **2AMARK** | Narratives regarding the company's specific attributes (e.g., size) or its environment or group of companies (e.g., industry or market). |
|  | Business role  B | Chief executive officer  General manager  Chief financial officer  Manager  Worker  Board of directors member  Board of advisors member  Non-executive role  Owner | **2BCEO**  **2BGM**  **2BCFO**  **2BMANAG**  **2BWORK**  **2BBDME**  **2BABME**  **2BNONEX**  **2BOWN** | The references to the role played in the company (Memili et al., 2015). For instance:  Top management team  Managerial roles  Workforce  Board of directors  Advisory forums  Non-executive chairwoman/chairman  Owner |
| **Shared vision**  **3** | Shared vision  A | Shared vision  Lack of shared vision | **3ASHAVIS**  **3ALACKSHAVIS** | Image of possibilities, optimism about family dreams, and a sense of hope (Boyatzis & Soler, 2012).  An inspiring vision for the future shared by the family members (Miller, 2014).  A "group member's genuine belief that they are working collaboratively toward a common purpose" (Lord 2015; p. 8).  The perception that a shared vision is lacking between family members. |
| **Trust 4** | Trust  A |  | **4ATRUST** | Perception of own vulnerability (Mayer et al., 1995) due to "the confident, positive expectation" (Lewicki et al., 1998, p.439) that another person facilitates our goals' achievement (Tjosvold et al., 2014). Perception of mutual identification is based on "shared values and commitments" (McAllister et al., 2006). |
|  | Trust components  B | Ability  Integrity  Benevolence | **4BABILIT**  **4BINTEG**  **4BBENEV** | "Ability is that group of skills, competencies,  and characteristics that enable a party to have influence within some specific domain." (Mayer et al.,1995, p. 717).  Perception that the other "person can manage the task at hand" (Stedham & Skaar, 2019, p.4).  Integrity is related to the perception of consistency of the party's past actions, credible communications about the trustee from other parties, belief that the trustee has a strong sense of justice, and the extent to which the party's actions are congruent with his or her words" (Mayer et al., 1995, p. 719).  "Benevolence is the perception of a positive orientation of  the trustee toward the trustor" (Mayer et al., 1995, p. 719).  "Benevolence is associated with trustors' perceptions that the trustee is a caring, warm person who is aware of and concerned with the needs and well-being of others" (Stedham & Skaar, 2019, p.4.). |
|  | Lack of trust  C | Lack of trust | **4CLACKTRU** | Trustor's perception that the trustee lacks ability, integrity, or benevolence (Mayer et al., 1995). Perception of mutual differences based on different values, culture, and commitments (McAllister et al., 2006). Trust declines "when positive expectations are disconfirmed" (Lewicki, 2006). |
|  | Trust repair  D | Trust repair | **4DTRUREP** | Perception of trust has been violated, and specific actions are needed to reestablish trust in relationships (e.g., excuses, apologies, denials, compensatory arrangements, contracts, monitoring, mitigate perceptions of harm, granting forgiveness; Lewicki & Brinsfield, 2017). |
| **Constructive conflict management**  **5** | Multilevel system  A | Informal family meetings  Family council  Managing committee  Board of directors  Board of advisors  Collaborators  Ownership  Family constitution  Teams | **5AFAMEET**  **5AFACOUN**  **5AMACOM**  **5ABOADIR**  **5ABOAADV**  **5ACOLLAT**  **5AOWN**  **5AFACONST**  **5ATEAM** | The different levels where the constructive conflict management may take place (e.g., informal family gatherings, family council, managing committees, the board of directors, the board of advisors, ownership, teams) (Borbély & Caputo, 2017; Mustakallio et al., 2002) and the use of unique tools of governance as Family Constitution or Family Protocol (Arteaga & Menendez-Requejo, 2017). |
|  | Perceived conflict  B | Perceived conflict  Lack of conflict  Conflict avoidance | **5BCONF**  **5BLACKCONF**  **5BCONFAVO** | Perception of differences, divergence, and controversies produce tensions, opposition, and interference (De Dreu & Gelfand, 2008; Deutsch, 1982/2011). Two or more family members are engaged in incompatible activities (Tjosvold et al., 2014). (Conflict). (Some typical examples of tensions in family firm context, e.g., change vs. continuity, founder vs. inheritor; Suddaby & Jaskiewicz, 2020).  The perception that there is no conflict between participants or that the organization is free of conflict (Tjosvold, 2008) (Lack of conflict).  Perception that the participants of a conflict are avoiding or eluding it (conflict avoidance). |
|  | Types of conflict  C | Task conflict  Relational conflict  Work-family conflict | **5CTASK**  **5CRELAT**  **5CWORKFAM** | "Task conflict is an awareness of differences in  viewpoints and opinions pertaining to a group task" (Jehn & Mannix, 2001, p.238).  Relationship conflict is "an awareness of interpersonal incompatibilities, including affective components such as feeling tension and friction" (Jehn & Mannix, 2001, p.238).  Work-family conflict is related to the perception of incompatibility between the roles exerted in the family and work domains (Lu et al., 2012). |
|  | Processes  D | Open-mindedness | **5DOMD** | Open, direct, and free dialogue between family firm members about their differences to clarify them and find a solution that integrates these thoughts and insights (Tjosvold et al., 2014; Deutsch, 2011).  "The committee's critical assessment of its assumptions, beliefs and prior actions, as well as its openness to new ideas" (Lord, 2015; p.11).  References to the four skills of open-minded debates: (1) Free development and expression of own ideas, (2) questioning and understanding other views, (3) integration and creation of new ideas, and (4) agreement and implementation of a solution (Tjosvold et al., 2014). |
|  |  | Close-mindedness | **5DCLOSE** | "New ideas are often rejected or overlooked because they conflict with the team members' rigid mental models, precluding anything novel  from being put into practice" (Harvey et al., 2019, p.1733). |
|  |  | Concurrence-seeking | **5DCONCSEEK** | The perception that members of family firms emphasize agreement in detriment of open discussion, which provokes no disagreements or  arguments. Also, they avoid an appraisal of alternative ideas and courses of action (Johnson & Johnson, 2009). |
|  | Third-party assistance  E | Third-party facilitation | **5ETHIRD** | Mention to people who assist in managing conflict in the family business constructively (e.g., non-family members, members of the board, external advisors, or consultants) (Lewicki et al., 2016; Qiu & Freel, 2020). |
|  | Succession  F | Succession | **5FSUCCESS** | Narratives regarding the process of transference of power, ownership, and managerial roles may lead to conflict between predecessors and successors (Filser et al., 2013). |
| **Innovation**  **6** | Innovativeness  A | Innovativeness  Collaborative innovation | **6AINNOVA**  **6ACOLLINNOV** | "A firm's tendency to engage in and support  new ideas, novelty, experimentation, and creative processes that may result in new products, services, or technological processes." (Lumpkin and Dess, 1996, p. 142).  Collaborative innovation is defined "as a form of inter-firm relationship that involves the exchange and sharing of resources such as financial capital, information, knowledge and technology with external parties in order to achieve innovation" (Feranita et al., 2017). |
|  | Risk-taking orientation  B | Risk-taking  Risk-avoidance | **6BRISKTAK**  **6BRISKAVOID** | Elusions to the participants' different preferences to invest a substantial amount of resources to achieve entrepreneurial goals (Kraiczy et al., 2012; Lumpkin & Dess, 1996), being risk-takers more willing and risk avoiders less willing to take these type of action. |
|  | External pressures  C |  | **6CEXT** | The perception of external trends and factors pushing to innovation activity (e.g., Social, Economic, Legal, Politic, Technology, environmental) (Cruz & Nordqvist, 2012). |
|  | Decision-making  pace  D | Agile decision-making  Slow decision-making  Blocked decision-making | **6DAGILE**  **6DSLOW**  **6DBLOCK** | The perception of the pace of decision-making processes related to innovation activities (Roessl et al., 2010). |

*References*

Arteaga, R., & Menéndez-Requejo, S. (2017). Family Constitution and Business Performance: Moderating Factors. *Family Business Review, 30*(4), 320-338. <https://doi.org/10.1177/0894486517732438>

Borbély, A., & Caputo, A. (2017). Approaching negotiation at the organizational level. *Negotiation and Conflict Management Research*, *10*(4), 306-323. <https://doi.org/10.1111/ncmr.12106>

Boyatzis, R. E., & Soler, C. (2012). Vision, leadership and emotional intelligence transforming family business. *Journal of Family Business Management*, *2*(1), 23-30. <https://doi.org/10.1108/20436231211216394>

Cruz, C., & Nordqvist, M. (2012). Entrepreneurial orientation in family firms: A generational perspective. *Small Business Economics*, *38*(1), 33-49. <https://doi.org/10.1007/s11187-010-9265-8>

De Dreu, C. K. W., & Gelfand, M. J. (Eds.). (2008). *The organizational frontiers series. The psychology of conflict and conflict management in organizatio*ns. Taylor & Francis Group/Lawrence Erlbaum Associates.

Deutsch, M. (1982/2011) Interdependence and Psychological Orientation. In: P.T. Coleman (ed.), *Conflict, Interdependence, and Justice*, Peace Psychology Book 247 Series, DOI 10.1007/978-1-4419-9994-8_11

Ensley, M. D., & Pearson, A. W. (2005). An exploratory comparison of the behavioral dynamics of top management teams in family and nonfamily new ventures: Cohesion, conflict, potency, and consensus. *Entrepreneurship Theory and Practice*, *29*(3), 267-284. <https://doi.org/10.1111/j.1540-6520.2005.00082.x>

Feranita, F., Kotlar, J., & De Massis, A. (2017). Collaborative innovation in family firms: Past research, current debates and agenda for future research. *Journal of Family Business Strategy, 8* (3), 137-156. <https://doi.org/10.1016/j.jfbs.2017.07.001>

Filser, M., Kraus, S., & Märk, S. (2013). Psychological aspects of succession in family business management. *Management Research Review*, 36(3), 256-277. <https://doi.org/10.1108/01409171311306409>

Harvey, J. F., Johnson, K. J., Roloff, K. S., & Edmondson, A. C. (2019). From orientation to behavior: The interplay between learning orientation, open-mindedness, and psychological safety in team learning. *Human Relations, 72*(11), 1726-1751. <https://doi.org/10.1177/0018726718817812>

Jehn, K. A., & Mannix, E. A. (2001). The dynamic nature of conflict: A longitudinal study of intragroup conflict and group performance. *Academy of management journal, 44*(2), 238-251. <https://doi.org/10.5465/3069453>

Johnson, D. W., & Johnson, R. T. (2009). Energizing learning: The instructional power of conflict. *Educational researcher*, *38*(1), 37-51. <https://doi.org/10.3102/0013189X08330540>

Knapp, J. R., Smith, B. R., Kreiner, G. E., Sundaramurthy, C., & Barton, S. L. (2013). Managing boundaries through identity work: The role of individual and organizational identity tactics. *Family Business Review*, *26*(4), 333-355. <https://doi.org/10.1177/0894486512474036>

Kraiczy, N. D., Hack, A., & Kellermanns, F. W. (2015). What makes a family firm innovative? CEO risk‐taking propensity and the organizational context of family firms. *Journal of Product Innovation Management*, *32*(3), 334-348. <https://doi.org/10.1111/jpim.12203>

Lansberg, I. (1999). *Succeeding generations: Realizing the dream of families in business*. Harvard Business Review Press.

Lewicki, R. J. (2006) Trust, Trust Development, and Trust Repair in M. Deutsch, P.T. Coleman & E. C. Marcus (Eds.) *The Handbook of Conflict Resolution Theory and Practice* (2^nd^ ed., pp. 92-119). Josey-Bass.

Lewicki, R. J., Barry, B., & Saunders, D. M. (2016). *Essentials of negotiation*. McGraw-Hill.

Lewicki, R. J., & Brinsfield, C. (2017). Trust repair. *Annual Review of Organizational Psychology and Organizational Behavior*, *4*, 287-313. <https://doi.org/10.1146/annurev-orgpsych-032516-113147>

Lewicki, R. J., McAllister, D.J. & Bies, R.J. (1998) Trust and distrust: New relationships and realities. *Academy of management Review*, *23*(3), 438-458. <https://doi.org/10.5465/amr.1998.926620>

Lord, M. (2015). Group learning capacity: the roles of open-mindedness and shared vision. *Frontiers in Psychology*, *6*, 150. <https://doi.org/10.3389/fpsyg.2015.00150>

Lu, J., Tjosvold, D., Shi, K., & Wang, B. (2012). Developing work–family balance through conflict management. *Asian Journal of Social Psychology*, *15*(2), 77-88. <https://doi.org/10.1111/j.1467-839X.2011.01363.x>

Lumpkin, G. T., & Dess, G. G. (1996). Clarifying the entrepreneurial orientation construct and linking it to performance. *Academy of management Review*, *21*(1), 135-172. <https://doi.org/10.5465/amr.1996.9602161568>

Mayer, R. C., Davis, J. H., & Schoorman, F. D. (1995). An integrative model of organizational trust. *Academy of management review*, *20*(3), 709-734. <https://doi.org/10.5465/amr.1995.9508080335>

McAllister, D. J., Lewicki, R. J., & Chaturvedi, S. (2006, August). Trust in developing relationships: from theory to measurement. In *Academy of Management Proceedings* (Vol. 2006, No. 1, pp. G1-G6). Briarcliff Manor, NY 10510: Academy of Management. <https://doi.org/10.5465/ambpp.2006.22897235>

Memili, E., Fang, H., Chrisman, J. J., & De Massis, A. (2015). The impact of small-and medium-sized family firms on economic growth. *Small Business Economics*, *45*(4), 771-785. <https://doi.org/10.1007/s11187-015-9670-0>

Miller, S. P. (2014). Next-generation leadership development in family businesses: the critical roles of shared vision and family climate. *Frontiers in Psychology*, *5*, 1335. <https://doi.org/10.3389/fpsyg.2014.01335>

Mustakallio, M., Autio, E., & Zahra, S. A. (2002). Relational and contractual governance in family firms: Effects on strategic decision making. *Family business review*, *15*(3), 205-222. <https://doi.org/10.1111/j.1741-6248.2002.00205.x>

Pearson, A. W., Carr, J. C., & Shaw, J. C. (2008). Toward a theory of familiness: A social capital perspective. *Entrepreneurship theory and practice*, *32*(6), 949-969. <https://doi.org/10.1111/j.1540-6520.2008.00265.x>

Qiu, H., & Freel, M. (2020). Managing Family-Related Conflicts in Family Businesses: A Review and Research Agenda. *Family Business Review, 33*(1), 90-113. [https://doi.org/10.1177/0894486519893223](https://doi.org/10.1177%2F0894486519893223)

Roessl, D., Fink, M., & Kraus, S. (2010). Are family firms fit for innovation? Towards an agenda for empirical research. *International Journal of Entrepreneurial Venturing*, 2 (3-4), 366-380. <https://doi.org/10.1504/IJEV.2010.037118>

Stedham, Y., & Skaar, T. B. (2019). Mindfulness, trust, and leader effectiveness: a conceptual framework. *Frontiers in psychology*, *10*. doi: [10.3389/fpsyg.2019.01588](https://dx.doi.org/10.3389%2Ffpsyg.2019.01588)

Suddaby, R., & Jaskiewicz, P. (2020). Managing traditions: A critical capability for family business success. *Family Business Review*, 33(3) 234–

243. <https://doi.org/10.1177/0894486520942611>

Tjosvold, D. (2008). The conflict‐positive organization: It depends upon us. *Journal of Organizational Behavior*, *29*(1), 19-28. <https://doi.org/10.1002/job.473>

Tjosvold, D., Wong, A. S., & Feng Chen, N. Y. (2014). Constructively managing conflicts in organizations. *Annual Review of Organizational Psychology and Organizational Behavior*. *1*(1), 545-568. <https://doi.org/10.1146/annurev-orgpsych-031413-091306>
